# A facile and one-pot synthesis of new tetrahydrobenzo[b]pyrans in water under microwave irradiation

**DOI:** 10.1186/s13065-019-0651-2

**Published:** 2019-11-26

**Authors:** Mandlenkosi Robert Khumalo, Surya Narayana Maddila, Suresh Maddila, Sreekantha B. Jonnalagadda

**Affiliations:** School of Chemistry & Physics, University of KwaZulu-Natal, Westville Campus, Chiltern Hills, Durban, 4000 South Africa

**Keywords:** Microwave irradiation, Multicomponent reactions, One-pot synthesis, Green synthesis, Benzopyrans

## Abstract

Eleven new tetrahydrobenzo[b]pyran derivatives were synthesized via a three component reaction of different aromatic aldehydes, methyl cyanoacetate and 1,3-cyclohexadione, with water as solvent under catalyst-free microwave irradiation. The structures of all the new molecules were well analysed and their structures established by using various spectral techniques (^1^H NMR, ^13^C NMR, ^15^N NMR and HRMS). Various advantages of reported protocol are the ease of preparation, short reaction times (10 min), aqueous solvent and excellent yields (89–98%). Additionally, this method provides a clean access to the desired products by simple workup.
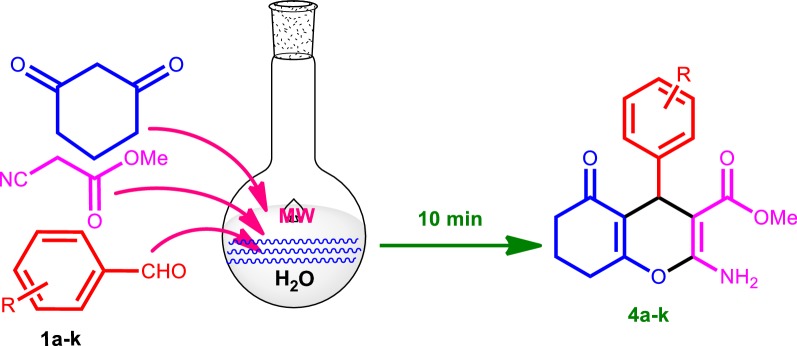

## Introduction

Multi component reaction (MCR) is an important technique for the effective and swift synthesis of a wide range of composite heterocyclic frameworks [1–3]. MCR is a distinctly focused approach for organic synthesis, because of their ability to make composite molecular functionality from the three or more starting materials through one-pot reaction [3–5] and for the creation of new C–C and C–O bonds [6]. Improvement in new multicomponent reactions with an environmentally benign perception has received ample attention due to the prospect of compliance with green chemistry principles [6, 7].

Reactions facilitated by microwave irradiation (MWI) have attracted significant attention, owing to the environmental benign operational simplicity and higher selectivity [8, 9]. MWI enhances the reaction rate by providing more energy to the reacting molecules and in many cases the reaction rate is 10- to 1000-fold faster than conventional heating [10, 11]. With advent of MWI, catalyst-free and solvent-free reactions have increased as they provide an opportunity to work with open vessels [12]. Furthermore, it circumvents the problems associated with higher-pressure conditions and offers a possibility for scaling-up the reaction under a moisture free environment [13]. Moreover, MWI offers other benefits including reduced reaction time, fast reaction optimization, mild reaction conditions, higher yields, reproducibility, lower solvent consumption and ease of synthesis of difficult compounds [14].

Heterocyclic frameworks have always presented an opportunity for the preparation of numerous privileged scaffolds with diverse biological activity [15–17]. Ease of MCR assembly and many sites for diversification helped mapping bioactive chemical space [7, 15–19]. Furthermore, new innovative and workable procedures for the synthesis of different heterocyclic molecules are always attractive. Benzopyran and its derivatives have appealed to the researchers from medicinal, organic, industrial and other chemical fields, due to their useful pharmacological or medicinal applications, such as anticancer [20], anti-HIV [21], antifungal [22], antiviral [23], anti-inflammatory [24], antimalarial [25] antioxidant [26] and antimicrobial [27] activities. They are also broadly used in perfumes, cosmetics, agrochemicals and in food as additives [28, 29]. Literature reveals reports for synthesis of benzopyrans using with various catalysts like hexamethylenetetraminebromine [30], magnetite-dihydrogen phosphate [31], Bmim[BF_4_] [32], PPA-SiO_2_ [33], Ca(OTf)_2_:Bu_4_NPF_6_ [34], phenylboronic acid [35] and H_6_P_2_W_12_O_62_·H_2_O [36], MWI/PEG [37] etc. Previously reported procedures come with various limitations, like use of expensive reagents/catalysts, toxic solvents, strict reaction conditions, low product yields, long reaction times and nonrecyclability of catalysts, which confine their scope in practical applications (details in Additional file 1: Table S1).

In our continuous quest for evolving facile and efficient approaches for the synthesis of diverse heterocycles via MCR methodologies [38–40], we have earlier reported the protocols for the synthesis of several heterocyclic biological active molecules [41–44]. The current work focus on the microwave irradiation approach for the first time, for the synthesis of a new series of benzopyran derivatives, through one-pot reaction of aromatic aldehyde, methyl cyanoacetate and 1,3-cyclohexadione using water as solvent.

## Experimental procedure

### General procedure for synthesis of tetrahydrobenzo[*b*]pyrans (**4a**–**k**)

**Fig. 1:**
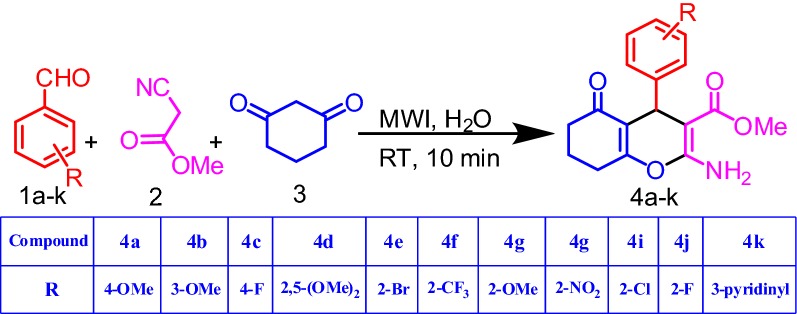
Three-component synthetic route for tetrahydrobenzo[b]pyran derivatives

A mixture of aromatic aldehyde (1 mmol), methyl cyanoacetate (1.1 mmol) and 1,3-cyclohexadione (1 mmol) were dissolved in water (5.0 mL) in a microwave vessel. Then, the mixture was microwave irradiated at 150 W for 10 min (Fig. 1). Thin layer chromatography (TLC) analysis was used to monitor the reaction progress. After completion of the reaction, the reaction mixture was cooled, filtered and washed with cold ice water. Further, the crude product was recrystallized by using ethanol to obtain pure product. Structures of all products were confirmed based on the spectral analysis with ^1^H NMR, ^15^N NMR (GHSQC), ^13^C NMR, ^19^F NMR, FTIR, and HRMS (instrumentation details in Additional file 1).

### Spectral data of representative compounds

#### Methyl 2-amino-4-(4-methoxyphenyl)-5-oxo-5,6,7,8-tetrahydro-4*H*-chromene-3-carboxylate (**4a**)

Mp.: 193–195 °C; ^1^H NMR (400 MHz, DMSO-d_6_) δ = 1.80–1.82 (m, 1H, CH_2_), 1.91–1.96 (m, 1H, CH_2_), 2.21–2.30 (m, 2H, CH_2_), 2.60–2.63 (m, 2H, CH_2_), 3.67 (s, 3H, OCH_3_), 3.87 (s, 3H, OCH_3_), 4.48 (s. 1H, CH), 6.75 (d, *J* = 8.64 Hz, 2H, ArH), 7.09 (d, *J* = 8.64 Hz, 2H, ArH), 7.50 (s, 2H, NH_2_); ^13^C NMR (100 MHz, DMSO-d_6_):19.85, 26.23, 30.62, 32.02, 36.29, 50.44, 53.09, 54.85, 55.73, 77.82, 79.11, 98.23, 113.22, 141.95, 123.91, 128.33, 133.51, 138.58, 154.55, 157.33, 159.23, 162.87, 163.57, 168.34, 196.02; ^15^N NMR (40.55 MHz, DMSO-d_6_) δ = 7.50 (s, 2H, NH_2_); FT-IR: 3397, 3302, 2944, 2843, 1725, 1689, 1583, 1509, 1429; HRMS of [C_18_H_19_NO_5_ + Na]^+^ (m/z): 352.1161; Calcd.: 352.1161.

#### Methyl 2-amino-4-(3-methoxyphenyl)-5-oxo-5,6,7,8-tetrahydro-4*H*-chromene-3-carboxylate (**4b**)

M.p.: 209–210 °C; ^1^H NMR (400 MHz, DMSO-d_6_) δ = 1.85–1.90 (m, 1H, CH_2_), 1.99–2.03 (m, 1H, CH_2_), 2.30–2.36 (m, 2H, CH_2_), 2.64–2.68 (m, 2H, CH_2_), 3.58 (s, 3H, OCH_3_), 3.75 (s, 3H, OCH_3_), 4.59 (s. 1H, CH), 6.73–6.78 (m, 3H, ArH), 7.18 (t, *J* = 8.68 Hz, 1H, ArH), 7.60 (s, 2H, NH_2_); ^13^C NMR (100 MHz, DMSO-d_6_):19.82, 26.24, 32.77, 36.25, 50.49, 54.76, 77.40, 110.60, 113.73, 116.78, 119.51, 128.93, 147.95, 158.80, 159.37, 164.15, 168.26, 196.03; ^15^N NMR (40.55 MHz, DMSO-d_6_) δ = 7.60 (s, 2H, NH_2_); FT-IR: 3404, 3280, 2946, 2836, 1682, 1665, 1594, 1510; HRMS of [C_18_H_19_NO_5_ + H]^+^ (m/z): 330.1763; Calcd.: 330.1766.

#### Methyl 2-amino-4-(4-fluorophenyl)-5-oxo-5,6,7,8-tetrahydro-4*H*-chromene-3-carboxylate (**4c**)

M.p.: 188–189 °C; ^1^H NMR (400 MHz, DMSO-d_6_) δ = 1.79–1.85 (m, 1H, CH_2_), 1.92–1.98 (m, 1H, CH_2_), 2.23–2.30 (m, 2H, CH_2_), 2.59–2.61 (m, 2H, CH_2_), 3.50 (s, 3H, OCH_3_), 4.53 (s. 1H, CH), 7.01 (d, *J* = 15.72 Hz, 2H, ArH), 7.15 (d, *J* = 3.08 Hz, 2H, ArH), 7.56 (s, 2H, NH_2_); ^13^C NMR (100 MHz, DMSO-d_6_): 19.80, 26.25, 30.65, 32.40, 36.23, 50.48, 53.33, 77.38, 101.91, 115.55, 116.73, 128.04, 128.08, 133.65, 133.75, 153.88, 159.23, 162.28, 163.40, 164.06, 168.17, 196.01; ^15^N NMR (40.55 MHz, DMSO-d_6_) δ = 7.56 (s, 2H, NH_2_); ^19^F NMR (376.58 MHz, DMSO): − 104.15; FT-IR: 3420, 3309, 2949, 1691, 1648, 1520, 1487; HRMS of [C_17_H_16_NO_4_F + Na]^+^ (m/z): 340.0992; Calcd.: 340.1008.

#### Methyl 2-amino-4-(2,5-dimethoxyphenyl)-5-oxo-5,6,7,8-tetrahydro-4*H*-chromene-3-carboxylate (**4d**)

M.p.: 222–223 °C; ^1^H NMR (400 MHz, DMSO-d_6_) δ = 1.90–2.03 (m, 3H, CH_3_), 2.29–2.33 (m, 2H, CH_2_), 2.51–2.56 (m, 2H, CH_2_), 3.58 (s, 3H, OCH_3_), 3.75 (s, 3H, OCH_3_), 3.77 (s, 3H, OCH_3_), 4.76 (s, 1H, CH), 6.17 (s, 2H, NH_2_), 6.64–6.67 (m, 1H, ArH), 6.72 (s, 1H, ArH), 6.90 (d, *J* = 3.08 Hz, 1H, ArH; ^13^C NMR (100 MHz, DMSO-d_6_): 20.36, 26.97, 31.44, 36.90, 50.78, 55.67, 56.59, 79.03, 111.99, 112.74, 116.05, 117.44, 122.63, 134.12, 149.73, 152.57, 153.14, 158.87, 163.48, 169.80, 196.56; ^15^N NMR (40.55 MHz, DMSO-d_6_) δ = 6.17 (s, 2H, NH_2_); FT-IR: 3391, 3270, 2952, 2839, 1727, 1685, 1590, 1428; HRMS of [C_19_H_21_NO_6_ + Na]^+^ (m/z): 382.1266; Calcd.: 382.1267.

#### Methyl 2-amino-4-(2-bromophenyl)-5-oxo-5,6,7,8-tetrahydro-4*H*-chromene-3-carboxylate (**4e**)

M.p.: 231–232 °C; ^1^H NMR (400 MHz, DMSO-d_6_) δ = 1.86–1.89 (m, 1H, CH_2_), 1.97–2.04 (m, 1H, CH_2_), 2.20–2.25 (m, 1H, CH_2_), 2.30–2.33 (m, 1H, CH_2_), 2.66 (t, *J* = 6.08 Hz, 2H, CH_2_), 3.51 (s, 3H,, OCH_3_), 4.89 (s. 1H, CH), 7.06 (t, *J* = 7.88 Hz, 1H, ArH), 7.21 (d, *J* = 7.8 Hz, 1H, ArH), 7.29 (t, *J* = 6.64 Hz, 1H, ArH), 7.47 (d, *J* = 6.8 Hz, 1H, ArH), 7.68 (s, 2H, NH_2_); ^13^C NMR (100 MHz, DMSO-d_6_): 19.81, 26.37, 30.65, 33.99, 36.39, 50.19, 76.74, 115.65, 123.18, 130.01, 132.47, 144.95, 153.41, 158.99, 163.94, 168.44, 195.65; ^15^N NMR (40.55 MHz, DMSO-d_6_) δ = 7.68 (s, 2H, NH_2_); FT-IR: 3409, 3292, 2949, 1724, 1689, 1645, 1514; HRMS of [C_17_H_16_BrNO_4_ + Na]^+^ (m/z): 400.0157; Calcd.: 400.0160.

#### Methyl 2-amino-4-(3-(trifluoromethyl)phenyl)-5-oxo-5,6,7,8-tetrahydro-4*H*-chromene-3-carboxylate (**4f**)

M.p.: 214–216 °C; ^1^H NMR (400 MHz, DMSO-d_6_) δ = 1.94–2.08 (m, 2H, CH_2_), 2.30–2.32 (m, 2H, CH_2_), 2.57–2.62 (m, 2H, CH_2_), 3.56 (s, 3H, OCH_3_), 5.32 (s. 1H, CH), 6.21 (s, 2H, NH_2_), 7.22 (t, *J* = 7.56 Hz, 2H, ArH), 7.38 (t, *J* = 7.4 Hz, 1H, ArH), 7.51 (d, *J* = 7.92 Hz, 1H, ArH); ^13^C NMR (100 MHz, DMSO-d_6_): 20.19, 27.00, 36.82, 50.70, 53.70, 80.66, 117.82, 126.30, 126.93, 126.97, 129.94, 130.62, 131.15, 144.70, 158.15, 162.90, 169.47, 196.26; ^15^N NMR (40.55 MHz, DMSO-d_6_) δ = 6.21 (s, 2H, NH_2_); ^19^F NMR (376.58 MHz, DMSO): − 53.68; FT-IR: 3500, 3415, 3308, 2948, 1689, 1650, 1526, 1307; HRMS of [C_18_H_16_F_3_NO_4_ + Na]^+^ (m/z): 390.0928; Calcd.: 390.0929.

#### Methyl 2-amino-4-(2-methoxyphenyl)-5-oxo-5,6,7,8-tetrahydro-4*H*-chromene-3-carboxylate (**4g**)

mp 235–237 °C; ^1^H NMR (400 MHz, DMSO-d_6_) δ = 1.76–1.95 (m, 2H, CH_2_), 2.14–2.25 (m, 2H, CH_2_), 2.55–2.59 (m, 2H, CH_2_), 3.45 (s, 3H, OCH_3_), 3.70 (s, 3H, OCH_3_), 4.60 (s. 1H, CH), 6.76–6.80 (m, 1H, ArH), 6.85 (t, *J* = 7.44 Hz, 1H, ArH), 7.05–7.07 (m, 1H, ArH), 7.12 (t, *J* = 5.76 Hz, 1H, ArH), 7.46 (s, 2H, NH_2_); ^13^C NMR (100 MHz, DMSO-d_6_): 20.49, 26.85, 31.40, 36.91, 39.99, 50.72, 56.09, 76.63, 112.38, 115.28, 120.11, 127.59, 131.50, 133.55, 158.21, 160.12, 164.63, 169.13, 196.32; ^15^N NMR (40.55 MHz, DMSO-d_6_) δ = 7.46 (s, 2H, NH_2_); FT-IR: 3389, 3251, 3192, 2946, 1683, 1637, 1529, 1460; HRMS of [C_18_H_19_NO_5_ + H]^+^ (m/z): 330.0929; Calcd.: 330.0937.

#### Methyl 2-amino-4-(2-nitrophenyl)-5-oxo-5,6,7,8-tetrahydro-4*H*-chromene-3-carboxylate (**4h**)

M.p.: 218–220 °C; ^1^H NMR (400 MHz, DMSO-d_6_) δ = 1.80–1.86 (m, 1H, CH_2_), 1.92–1.98 (m, 1H, CH_2_), 2.13–2.20 (m, 1H, CH_2_), 2.25–2.30 (m, 1H, CH_2_), 2.61 (t, *J* = 5.88 Hz, 2H, CH_2_), 3.38 (s, 3H, OCH_3_), 5.32 (s. 1H, CH), 7.29–7.34 (m, 2H, ArH), 7.53–7.57 (m, 1H, ArH), 7.71 (s, 2H, NH_2_), 7.73 (d, *J* = 6.92 Hz, 1H, ArH); ^13^C NMR (100 MHz, DMSO-d_6_): 19.73, 26.41, 28.57, 36.29, 50.41, 76.37, 115.40, 123.81, 126.97, 130.23, 132.80, 140.65, 148.74, 159.16, 164.48, 168.13, 195.80; ^15^N NMR (40.55 MHz, DMSO-d_6_) δ = 7.71 (s, 2H, NH_2_); FT-IR: 3518, 3401, 3292, 2947, 1688, 1649, 1519, 1351; HRMS of [C_17_H_16_N_2_O_6_ + Na]^+^ (m/z): 367.0908; Calcd.: 367.0906.

#### Methyl 2-amino-4-(2-chlorophenyl)-5-oxo-5,6,7,8-tetrahydro-4*H*-chromene-3-carboxylate (**4i**)

M.p.: 210–213 °C; ^1^H NMR (400 MHz, DMSO-d_6_) δ = 1.87–1.95 (m, 2H, CH_2_), 2.23–2.26 (m, 2H, CH_2_), 2.46–2.51 (m, 2H, CH_2_), 3.49 (s, 3H, OCH_3_), 4.94 (s. 1H, CH), 6.13 (s, 2H, NH_2_), 6.97 (t, *J* = 7.72 Hz, 1H, ArH), 7.06 (t, *J* = 7.36 Hz, 1H, ArH) 7.16 (d, *J* = 6.56 Hz, 1H, ArH), 7.21 (d, *J* = 7.68 Hz, 1H, ArH);^13^C NMR (100 MHz, DMSO-d_6_): 20.24, 26.97, 32.99, 36.87, 50.78, 79.19, 116.17, 126.20, 127.34, 129.84, 132.11, 133.67, 142.01, 158.36, 163.45, 169.52, 196.39; ^15^N NMR (40.55 MHz, DMSO-d_6_) δ = 6.13 (s, 2H, NH_2_); FT-IR: 3453, 3392, 2954, 1721, 1687, 1603, 1492; HRMS of [C_17_H_16_ClNO_4_ + Na]^+^ (m/z): 356.1169; Calcd.: 356.1168.

#### Methyl 2-amino-4-(2-fluorophenyl)-5-oxo-5,6,7,8-tetrahydro-4*H*-chromene-3-carboxylate (**4j**)

M.p.: 217–219 °C; ^1^H NMR (400 MHz, DMSO-d_6_) δ = 1.96–2.05 (m, 2H, CH_2_), 2.31–2.35 (m, 2H, CH_2_), 2.56–2.60 (m, 2H, CH_2_), 3.60 (s, 3H, OCH_3_), 4.84 (s, 1H, CH), 6.21 (s, 2H, NH_2_), 6.88–6.93 (m, 1H, ArH), 7.01 (t, *J* = 6.28 Hz, 1H, ArH) 7.08–7.11 (m, 1H, ArH), 7.29–7.33 (m, 1H, ArH); ^13^C NMR (100 MHz, DMSO-d_6_): 20.28, 26.91, 29.77, 30.93, 36.80, 50.88, 53.54, 78.91, 115.30, 123.40, 123.43, 124.94, 124.98, 127.76, 129.11, 131.40, 131.45, 135.29, 135.39, 146.53, 146.61, 158.55, 160.03, 162.50, 163.63, 169.47, 196.45; ^15^N NMR (40.55 MHz, DMSO-d_6_) δ = 6.21 (s, 2H, NH_2_); ^19^F NMR (376.58 MHz, DMSO): − 53.51; FT-IR: 3420, 3309, 2949, 1691, 1648, 1520, 1487; HRMS of [C_17_H_16_FNO_4_ + Na]^+^ (m/z): 340.0956; Calcd.: 340.0961.

#### Methyl 2-amino-4-(pyridine-3-yl)-5-oxo-5,6,7,8-tetrahydro-4*H*-chromene-3-carboxylate (**4k**)

M.p.: 222–223 °C; ^1^H NMR (400 MHz, DMSO-d_6_) δ = 1.81–1.86 (m, 1H, CH_2_), 1.93–1.97 (m, 1H, CH_2_), 2.23–2.31 (m, 2H, CH_2_), 2.60–2.64 (m, 2H, CH_2_), 3.50 (s, 3H, OCH_3_), 4.52 (s, 1H, CH), 7.21–7.25 (m, 1H, ArH), 7.46–7.49 (m, 1H, ArH) 7.08–7.11 (m, 1H, ArH), 7.62 (s, 2H, NH_2_), 8.28 (d, *J *= 4.72 Hz, 1H, ArH), 8.38 (d, *J *= 1.96 Hz, 1H, ArH);^13^C NMR (100 MHz, DMSO-d_6_): 19.79, 26.26, 31.18, 36.16, 50.54, 76.62, 115.71, 123.28, 134.83, 141.71, 146.97, 149.06, 159.20, 164.53, 167.99, 196.04; ^15^N NMR (40.55 MHz, DMSO-d_6_) δ = 7.62 (s, 2H, NH_2_); FT-IR: 3372, 2996, 1671, 1530, 1362, 1293; HRMS of [C_16_H_16_N_2_O_4_ + Na]^+^ (m/z): 323. 1009; Calcd.: 323.1008.

## Results and discussion

### Reaction optimization

Based on preliminary studies, 2-methoxy benzaldehyde (1 mmol), methyl cyanoacetate (1.1 mmol) and 1,3-cyclohexadione (1 mmol) were identified as ideal for the multicomponent reaction. The effect of solvent on the reaction were assessed under MWI and conventional heating conditions. The results using different non-polar, aprotic and protic solvents under conventional heating and MWI conditions are summarised in Table 1. No reaction occurred in absence of solvent, under conventional, MWI, RT or reflux conditions. Non-polar solvents like n-hexane and toluene failed to produce any product, even after long reaction time at RT (Table 1, entries 3 and 4). However, the presence of polar aprotic solvents, DMF, THF and acetonitrile revealed a trace of anticipated product (Table 1, entries 5–7), under both conventional and MWI conditions. With polar protic solvents, MeOH, EtOH and water offered, good to excellent yields with both conventional heating and MWI, but MWI proved better in terms of yield and reaction times (Table 1, entries 8–10). The reason for the low yield, when using conventional heating could also be likely due to the steric demand for 2-substituted aromatics.

**Table 1:** Yields of benzopyran (**4a**) under diverse conventional heating and MWI conditions

Entry	Solvent	Condition	Conventional		MWI	
			Time (h)	Yield^a^ (%)	Time (h)	Yield^a^ (%)
**1**	–	R.T	12.0	**–**	6.0	**–**
**2**	**–**	Heat	10.0	**–**	6.0	**–**
**3**	*n*-Hexane	R.T	10.0	**–**	4.0	**–**
**4**	Toluene	R.T	10.0	**–**	4.0	**–**
**5**	THF	R.T	5.0	5	2.5	13
**6**	CH_3_CN	R.T	5.5	6	3.0	10
**7**	DMF	R.T	6.0	9	2.5	15
**8**	MeOH	R.T	3.5	67	2.5	71
**9**	EtOH	R.T	2.5	71	0.5	84
**10**	H_2_O	R.T	3.0	79	0.20	98

All products were characterized by ^1^HNMR, ^13^C NMR, ^15^N NMR and HR-MS spectral data

^a^Isolated yields; –: no reaction

The polar protic solvents, when microwave irradiated generate more dipole moments and their dipole moments effectively align with the external electric field. Based on the impressive yields and short reaction times, the MWI procedure with environmentally benign water proved to be ideal. Hence, MWI with water was used for the further studies.

Under the optimized reaction conditions, the MWI approach was applied for preparation of series of benzopyran derivatives, employing different aromatic aldehydes and methyl cyanoacetate and 1,3-cyclohexadione. Table 2 summarizes the results. All the aldehydes reacted smoothly to afford the desired target molecules without any side products. The electronic nature of substituents on the aromatic aldehyde ring did not show any effect on the yield or reaction rate. Both electron withdrawing and donating substituents on the aldehyde ring gave the excellent yield for the respective product. ^1^H NMR, ^13^C NMR, ^15^N NMR, ^19^F NMR, HRMS and IR spectral data were used to evaluate the structures of all the newly synthesised molecules (4a–k). Spectra of all the compounds are incorporated in Additional file 1. The HMBC interactions of trial reaction 4g are shown in Additional file 1: Figure S1. In the 1H NMR spectra, the individual singlets peaks at δ = 3.45, 3.70, 4.60 and 7.46 indicate the presence of –OCH_3_, –CH and –NH_2_ protons. The selected HMBC interactions of 4 g are definite proof for the product formation. The –CH proton in the benzo pyran ring was assigned to the peak at δ = 4.60 and it further interacts with carbon atoms (C-3, C-9, C-1a, C-2a, C-10, C-2, C-11, C-5) at δ = 76.63, 115.28, 133.55, 158.21, 160.12, 164.63, 169.13 and 196 ppm respectively. The singlet at δ = 7.46 was identified to the –NH_2_ proton in the benzo pyran ring (Additional file 1: Figure S2).

**Table 2:** Preparation of tetrahydrobenzo[b]pyran derivatives in water as solvent using MWI

Entry	R	Product	Yield (%)
1a	4-OMe	**4a**	96
1b	3-OMe	**4b**	92
1c	4-F	**4c**	94
1d	2,5-(OMe)_2_	**4d**	90
1e	2-Br	**4e**	93
1f	2-CF_3_	**4f**	89
1g	2-OMe	**4** **g**	98
1h	2-NO_2_	**4** **h**	94
1i	2-Cl	**4i**	89
1j	2-F	**4j**	92
1k	3-Pyridinyl	**4k**	95

New compounds/no literature for bps available

**Fig. 2:**
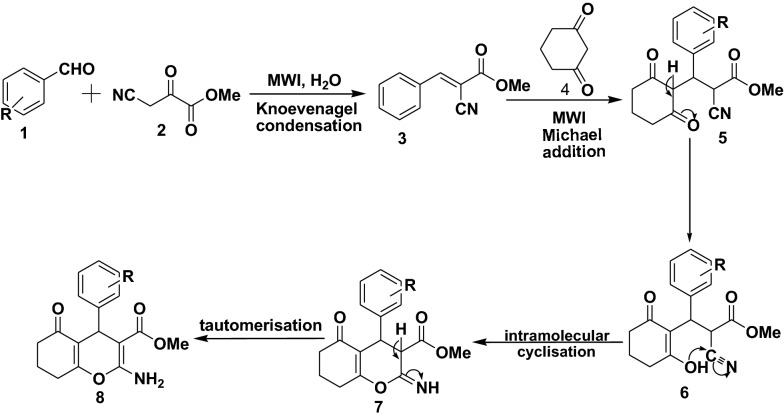
Proposed reaction mechanism for tetrahydrobenzo[b]pyrans derivatives

Although, no reaction intermediates could be identified, based on the reaction products and the literature reports, the probable mechanism for the synthesis of benzopyran derivatives under MWI is described (Fig. 2). Initially, an aromatic aldehyde (1) react with methyl cyanoacetate (2) via Knoevenagel condensation to afford an intermediate, cyanophenylacrylate (3) [45, 46]. The intermediate reacts with the active methylene moiety in (4) via Michael addition, through the electrophilic C=C bond to afford transient intermediate (5) [47]. Finally, the intermediate (6) undergoes intramolecular cyclisation followed by tautomerisation, to afford its respective benzopyran derivative.

## Conclusion

The MWI facilitated three-component synthesis of eleven novel tetrahydrobenzo[b]pyrans through one-pot reaction with water as solvent proved an expedient technique. It is applicable for the archive preparation of benzopyran systems in excellent yields, with no need for catalysts or organic solvents. This method offers extensive applications in the field of diversity-oriented synthesis, drug discovery, combinatorial chemistry and scaled-up preparations.

## Supplementary information

**Additional file 1.** Additional instrumental details, spectral data and details of product yields. **Figure S1:** Selected HMBC interactions of –CH & a (1–6) protons of 4**g**. **Figure S2:** ^1^H and ^13^C chemical shift of compound 4**g**. **Table S1:** Effect of various conditions for the synthesis of benzopyrans in presence of several catalysts.

## Data Availability

A Additional file is provided incorporating the additional data. S1—All instruments’ details, S2—Spectral information of the all synthesized compounds plus the 2D NMR data for 4g compound, UV–Visible spectrum of benzopyran and details of product yields in Additional file 1: Table S1.

## Abbreviations

^1^H NMR: proton nuclear magnetic resonance
^13^C NMR: carbon-13 nuclear magnetic resonance
^15^N NMR: nitrogen-15 nuclear magnetic resonance
^19^F NMR: fluorine-19 nuclear magnetic resonance
C–C: carbon–carbon bond
C–O: carbon–oxygen bond
CH3CN: acetonitrile
Ca(OTf)_2_:Bu_4_NPF_6_: calciumtriflate and tetra-butyl hexafloroammoniumphosphate
DMF: *N*,*N*-dimethylmethanamide
DMSO-d_6_: deuterated dimethyl sulfoxide
EtOH: ethanol
FT-IR: Fourier transform infrared spectroscopy
MeOH: methanol
MWI: microwave irradiation
MCR: multi component reaction
THF: tetrahydrofuran

## References

[1] Maddila S, Gangu KK, Maddila SN, Jonnalagadda SB (2017). A facile, efficient and sustainable chitosan/CaHAp catalyst and one-pot synthesis of novel 2,6-diamino-pyran-3,5-dicarbonitriles. Mol Divers.

[2] Shabalala S, Maddila S, van Zyl WE, Jonnalagadda SB (2017). An innovative efficient method for the synthesis of 1,4-dihydropyridines using Y_2_O_3_ loaded on ZrO_2_ as catalyst. ACS Ind Eng Chem Res.

[3] Gangu KK, Maddila S, Jonnalagadda SB (2017). Synthesis, structure and properties of new Mg(II)-metal-organic framework and its prowess as catalyst in the production of 4H-pyrans. ACS Ind Eng Chem Res.

[4] Moodley V, Maddila S, Jonnalagadda SB, van Zyl WE (2017). Synthesis of triazolidine-3-one derivatives through the nanocellulose/hydroxyapatite-catalyzed reaction of aldehydes and semicarbazide. New J Chem.

[5] Bhaskaruni SVHS, Maddila S, van Zyl WE, Jonnalagadda SB (2017). RuO_2_/ZrO_2_ as an efficient reusable catalyst for the facile, green, one-pot synthesis of novel functionalized halopyridine derivatives. Catal Commun.

[6] Shabalala S, Maddila S, van Zyl WE, Jonnalagadda SB (2016). A facile, efficacious and reusable Sm_2_O_3_/ZrO_2_ catalyst for the novel synthesis of functionalized 1,4-dihydropyridine derivatives. Catal Commun.

[7] Gangu KK, Maddila S, Maddila SN, Jonnalagadda SB (2017). Novel iron doped calcium oxalates as promising heterogeneous catalysts for one-pot multi-component synthesis of pyranopyrazoles. RSC Adv.

[8] Maddila S, Gangu KK, Maddila SN, Jonnalagadda SB (2017). Recent advances in the synthesis of pyrazole derivatives by multicomponent reaction. Curr Org Synth.

[9] Varma RS (1999). Solvent-free organic syntheses, using supported reagents and microwave irradiation. Green Chem.

[10] Moloi S, Maddila S, Jonnalagadda SB (2017). Microwave irradiated one-pot synthesis of quinoline derivatives catalyzed by triethyl amine. Res Chem Intermed.

[11] Kappe CO (2008). Microwave dielectric heating in synthetic organic chemistry. Chem Soc Rev.

[12] Taher A, Nandi D, Islam RU, Choudhary M, Mallick K (2015). Microwave assisted azide–alkyne cycloaddition reaction using polymer supported Cu(I) as a catalytic species: a solventless approach. RSC Adv.

[13] Sharma A, Appukkuttan P, der Eycken EV (2012). Microwave-assisted synthesis of medium-sized heterocycles. Chem Commun.

[14] Dolzhenko AV, Kalinina SA, Kalinin DV (2013). A novel multicomponent microwave-assisted synthesis of 5-aza-adenines. RSC Adv.

[15] Gorle S, Maddila S, Maddila SN, Naicker K, Singh M, Singh P, Jonnalagadda SB (2017). Synthesis, molecular docking study and in vitro anticancer activity of tetrazole linked benzochromene derivatives. Anticancer Agents Med Chem.

[16] Maddila S, Naicker K, Gorle S, Rana S, Yalagala K, Maddila SN, Singh M, Singh P, Jonnalagadda SB (2016). New pyrano[2,3-d:6,5-d’]dipyrimidine derivatives—synthesis, in vitro cytotoxicity activity and computational studies. Anticancer Agents Med Chem.

[17] Maddila SN, Maddila S, Gangu KK, van Zyl WE, Jonnalagadda SB (2017). Sm_2_O_3_/Fluoroapatite as a reusable catalyst for the facile, green, one-pot synthesis of triazolidine-3-thione derivatives under aqueous conditions. J Fluorine Chem.

[18] Shabalala N, Maddila S, Jonnalagadda SB (2016). Catalyst-free, one-pot, four-component green synthesis of functionalized 1-(2-fluorophenyl)-1,4-dihydropyridines under ultrasound irradiation. New J Chem.

[19] Maddila SN, Maddila S, van Zyl WE, Jonnalagadda SB (2016). Swift and green protocol for one-pot synthesis of pyrano[2,3-c]pyrazole-3-carboxylates with RuCaHAp as catalyst. Curr Org Chem.

[20] Singh S, Ahmad A, Raghuvanshi DS, Hasanain M, Agarwal K, Dubey V, Fatima K, Alam S, Sarkar J, Luqman S, Khan F, Tandon S, Gupta A (2016). Synthesis of 3,5-dihydroxy-7,8-dimethoxy-2-(4-methoxyphenyl)benzopyran-4-one derivatives as anticancer agents. Bioorg Med Chem Lett.

[21] Singh IP, Bodiwala HS (2010). Recent advances in anti-HIV natural products. Nat Prod Rep.

[22] Nawaz M, Abbasi MW, Hisaindee S (2016). Synthesis, characterization, anti-bacterial, anti-fungal and nematicidal activities of 2-amino-3-cyanochromenes. J Photochem Photobiol B.

[23] Raic-Malic S, Tomaskovic L, Mrvos-Sermek D, Prugovecki B, Cetina M, Grdisa M, Pavelic K, Mannschreck A, Balzarini J, De Clercq E, Mintas M (2004). Spirobipyridopyrans, spirobinaphthopyrans, indolinospiropyridopyrans, indolino spiro naphthopyrans and indolinospironaphtho-1,4-oxazines: synthesis, study of X-ray crystal structure, antitumoral and antiviral evaluation. Bioorg Med Chem.

[24] Hasan SM, Alam MM, Husain A, Khanna S, Akhtar M, Zaman MS (2009). Synthesis of 6-aminomethyl derivatives of benzopyran-4-one with dual biological properties: anti-inflammatory-analgesic and antimicrobial. Eur J Med Chem.

[25] Devakaram R, StC D, Black KT, Andrews GM, Fisher R, Davis N Kumar (2011). Synthesis and antimalarial evaluation of novel benzopyrano[4,3-b]benzopyran derivatives. Bioorg Med Chem.

[26] Grazul M, Kufelnicki A, Wozniczka M, Lorenz I, Mayer P, Jozwiak A, Czyz M, Budzisz E (2012). Synthesis, structure, electrochemical properties, cytotoxic effects and antioxidant activity of 5-amino-8-methyl-4H-benzopyran-4-one and its copper(II) complexes. Polyhedron.

[27] Ronad PM, Noolvi MN, Sapkal S, Dharbhamulla S, Maddi VS (2010). Synthesis and antimicrobial activity of 7-(2-substituted phenylthiazolidinyl)-benzopyran-2-one derivatives. Eur J Med Chem.

[28] Stevens JC, Merritt DJ, Flematti GR, Ghisalberti EL, Dixon KW (2007). Seed germination of agricultural weeds is promoted by the butenolide 3-methyl-2H-furo[2,3-c]pyran-2-one under laboratory and field conditions. Plant Soil.

[29] Parker SR, Cutler HG, Jacyno JM, Hill RA (1997). Biological activity of 6-pentyl-2h-pyran-2-one and its analogs. J Agric Food Chem.

[30] Jin TS, Wang AQ, Wang X, Zhang JS, Li TS (2004). A clean one-pot synthesis of tetrahydrobenzo[b]pyran derivatives catalyzed by hexadecyl trimethyl ammonium bromide in aqueous media. Synlett.

[31] Moshtaghin HS, Zonoz FM (2017). Preparation and characterization of magnetite-dihydrogen phosphate as a novel catalyst in the synthesis of tetrahydrobenzo[b]pyrans. Mater Chem Phys.

[32] Peng Y, Song G, Huang F (2005). Tetramethylguanidine-[bmim][BF_4_]. An efficient and recyclable catalytic system for one-pot synthesis of 4H-pyrans. Monatshefte für Chemie.

[33] Davoodnia A, Allameh S, Fazil S, Tavakoli-Hoseini N (2011). One-pot synthesis of 2-amino-3-cyano-4-arylsubstituted tetrahydrobenzo[b]pyrans catalysed by silica gel-supported polyphosphoric acid (PPA-SiO_2_) as an efficient and reusable catalyst. Chem Pap.

[34] Yaragorla S, Saini P, Singh G (2015). Alkaline earth metal catalyzed cascade, one-pot, solvent-free, and scalable synthesis of pyranocoumarins and benzo[b]pyrans. Tetrahedron Lett.

[35] Nemouchi S, Boulcina R, Carboni B, Debache A (2012). Phenylboronic acid as an efficient and convenient catalyst for a three-component synthesis of tetrahydrobenzo[b]pyrans. C R Chim.

[36] Heravi MM, Jani BA, Derikvand F, Bamoharram FF, Oskooie HA (2008). Three component, one-pot synthesis of dihydropyrano [3,2-c] chromene derivatives in the presence of H_6_P_2_W_18_O_62_18H_2_O as a green and recyclable catalyst. Catal Commun.

[37] Feng C, Wang Q, Lu C, Yang G, Chen Z (2012). Green synthesis of tetrahydrobenzo[b]pyrans by microwave assisted multi-component one-pot reactions in PEG-400. Comb Chem High Throughput Screen.

[38] Maddila SN, Maddila S, van Zyl WE, Jonnalagadda SB (2016). Ce-V/SiO_2_ catalyzed cascade for C-C and C-O bond activation: green one-pot synthesis of 2-amino-3-cyano-H-pyrans. ChemistryOpen.

[39] Maddila SN, Maddila S, van Zyl WE, Jonnalagadda SB (2016). Ag/SiO_2_ as a recyclable catalyst for the facile green synthesis of 3-methyl-4-(phenyl)-methylene-isoxazole-5(4H)-ones. Res Chem Intermed.

[40] Maddila SN, Maddila S, van Zyl WE, Jonnalagadda SB (2015). Mn doped ZrO_2_ as a green, efficient and reusable heterogeneous catalyst for the multicomponent synthesis of pyrano[2,3-d]-pyrimidine derivatives. RSC Adv.

[41] Maddila S, Naicker K, Momin M, Rana S, Gorle S, Maddila SN, Yalagala K, Singh M, Jonnalagadda SB (2016). Novel 2-(1-(substitutedbenzyl)-1H-tetrazol-5-yl)-3-phenylacrylonitrile derivatives—synthesis, in vitro antitumor activity and computational studies. Med Chem Res.

[42] Gorle S, Maddila S, Chokkakula S, Lavanya P, Singh M, Jonnalagadda SB (2016). Synthesis, biological activity of pyrimidine linked with morpholinophenyl derivatives. J Heterocycl Chem.

[43] Maddila S, Gorle S, Seshadri N, Lavanya P, Jonnalagadda SB (2016). Synthesis, antibacterial and antifungal activity of novel benzothiazole pyrimidine derivatives. Arab J Chem.

[44] Maddila S, Gorle S, Singh M, Lavanya P, Jonnalagadda SB (2013). Synthesis and anti-inflammatory activity of fused 1,2,4-triazolo-[3,4-b][1,3,4]-thiadiazole derivatives of phenothiazine. Lett Drug Des Discov.

[45] Maddila SN, Maddila S, Khumalo M, Bhaskaruni SVHS, Jonnalagadda SB (2019). An eco-friendly approach for synthesis of novel substituted4H-chromenes in aqueous ethanol under ultra-sonication with 94% atom economy. J Mol Struct.

[46] Balalaie S, Ahmadi MS, Bararjanian M (2007). Tetra-methyl ammonium hydroxide: an efficient and versatile catalyst for the one-pot synthesis of tetrahydrobenzo[b]pyran derivatives in aqueous media. Catal Commun.

[47] Rong L, Li X, Wang H, Shi D, Tu S, Zhuang Q (2006). Efficient synthesis of tetrahydrobenzo[b]pyrans under solvent-free conditions at room temperature. Synth Commun.

