# Supplementary material for: A facile and one-pot synthesis of new tetrahydrobenzo[b]pyrans in water under microwave irradiation
Source: BMC Chem. 2019 Nov 26;13(1):132. doi: 10.1186/s13065-019-0651-2 (PMC6878704; doi:10.1186/s13065-019-0651-2)
Supplement: Supplementary file 1 — Additional file 1. Additional instrumental details, spectral data and details of product yields. Figure S1: Selected HMBC interactions of –CH & a (1–6) protons of 4g. Figure S2: 1H and 13C chemical shift of compound 4g. Table S1: Effect of various conditions for the synthesis of benzopyrans in presence of several catalysts. [file 13065_2019_651_MOESM1_ESM.doc]

**Facile one-pot synthesis of new tetrahydrobenzo[b]pyrans in water using microwave irradiation**

Mandlenkosi Robert Khumalo, Surya Narayana Maddila, Suresh Maddila and Sreekantha B Jonnalagadda*****

*School of Chemistry & Physics, University of KwaZulu-Natal, Westville Campus,

Chiltern Hills, Durban-4000, South Africa.

***Corresponding Author:** Prof. Sreekantha B. Jonnalagadda

School of Chemistry & Physics,

University of KwaZulu-Natal,

Durban 4000, South Africa.

Tel.: +27 31 2607325,

Fax: +27 31 2603091

E-mail address: [**jonnalagaddas@ukzn.ac.za**](mailto:jonnalagaddas@ukzn.ac.za)

| **Contents** | **Pages** |
| --- | --- |
| **All the Instruments details– S1** | **2** |
| **All spectral information of the synthesized compounds – S2** | **3-35** |
| **2D NMR data for 4g compound** | **23** |
| **UV-Visible spectrum of BenzoPyran** | **36** |
| **Details of Product yields in Table S1** | **37** |

**Materials and instruments:**

All reagents and chemicals used were analytical grade used without further purification and purchased from Sigma-Aldrich. MW-assisted reaction was carried out in a CEM-908010, 300-W bench mate model laboratory MW reactor. The MW power was 150 W for the reaction at 80 °C with high stirring. Product formation was checked with thin layer chromatography (TLC) coated with silica gel 60 F254. High-resolution mass data were obtained using a Bruker micro TOF-Q II ESI instrument operating at ambient temperature. A Bruker AMX 400 MHz NMR spectrometer was used to record the ^1^H NMR, ^15^N NMR (GHSQC), ^13^C NMR spectral values of the products in DMSO-d_6_. TMS served as internal standard for reporting the chemical shifts in d (ppm). Infrared (IR) spectra were recorded on a Perkin Elmer Precisely equipped with a Universal ATR sampling accessory using a diamond crystal. The powdered material was placed on the crystal and a force of 120 psi was applied to ensure proper contact between the material and the crystal. The spectra were analyzed using Spectrum 100 software.


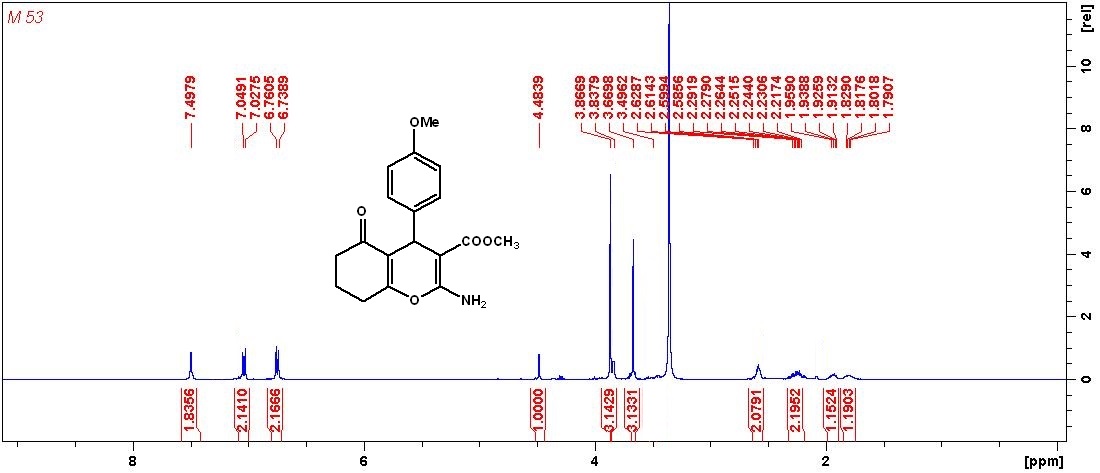


^1^H NMR spectra of compound **4a**

**
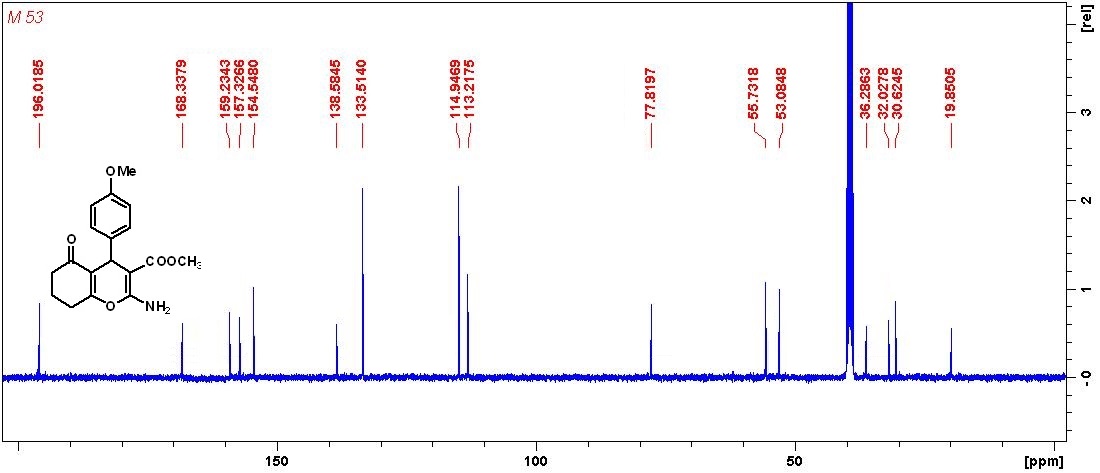
**

^13^C NMR spectra of compound **4a**


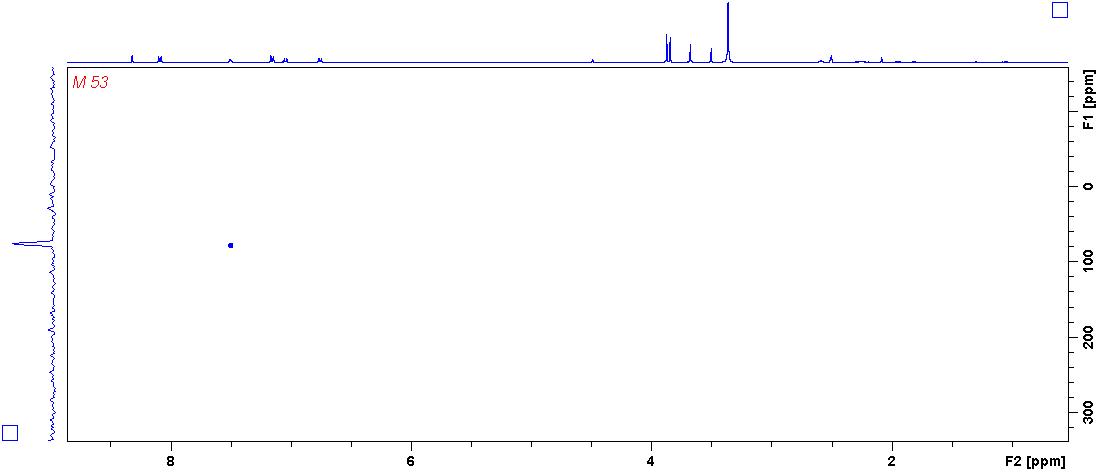


^15^N NMR spectra of compound **4a**

IR spectrum of compound **4a**

HRMS spectra of compound **4a**


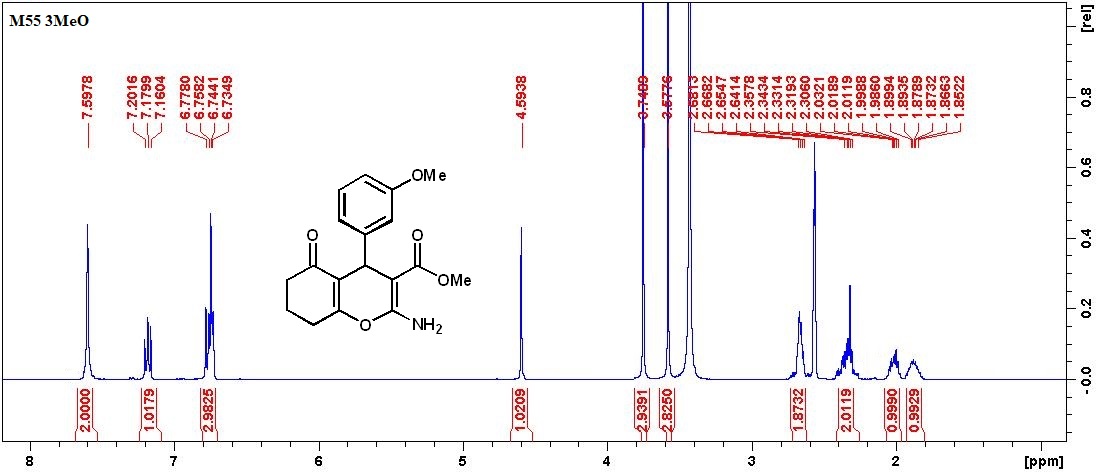


^1^H NMR spectra of compound **4b**

**
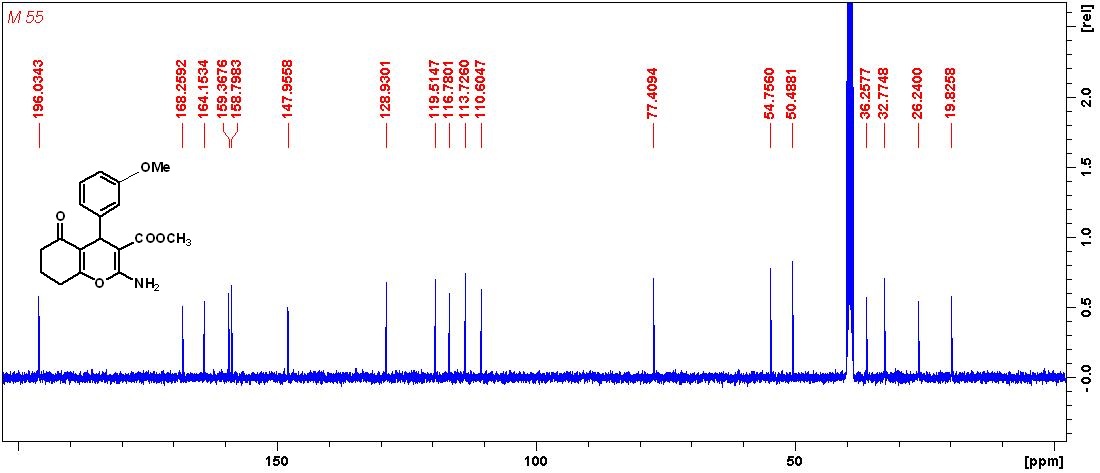
**

^13^C NMR spectra of compound **4b**

**
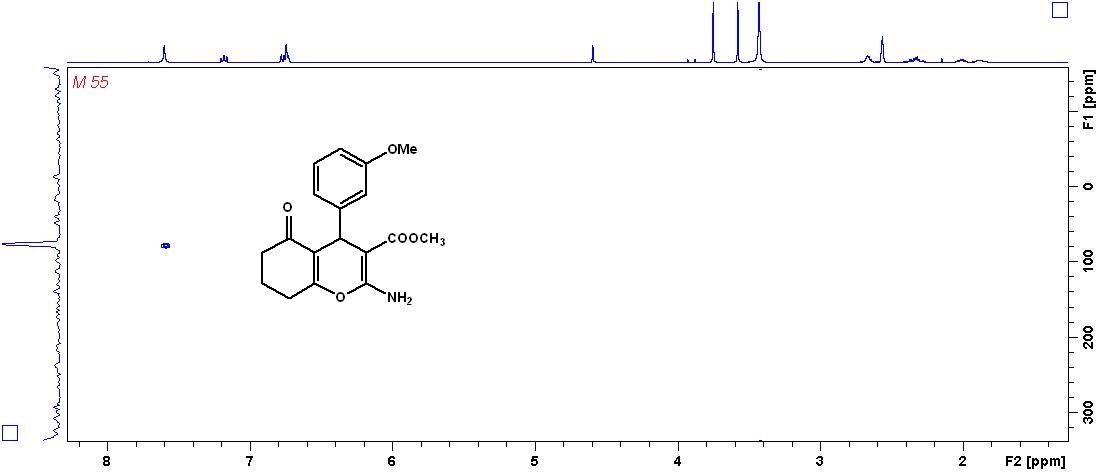
**

^15^N NMR spectra of compound **4b**

IR spectrum of compound **4b**

**
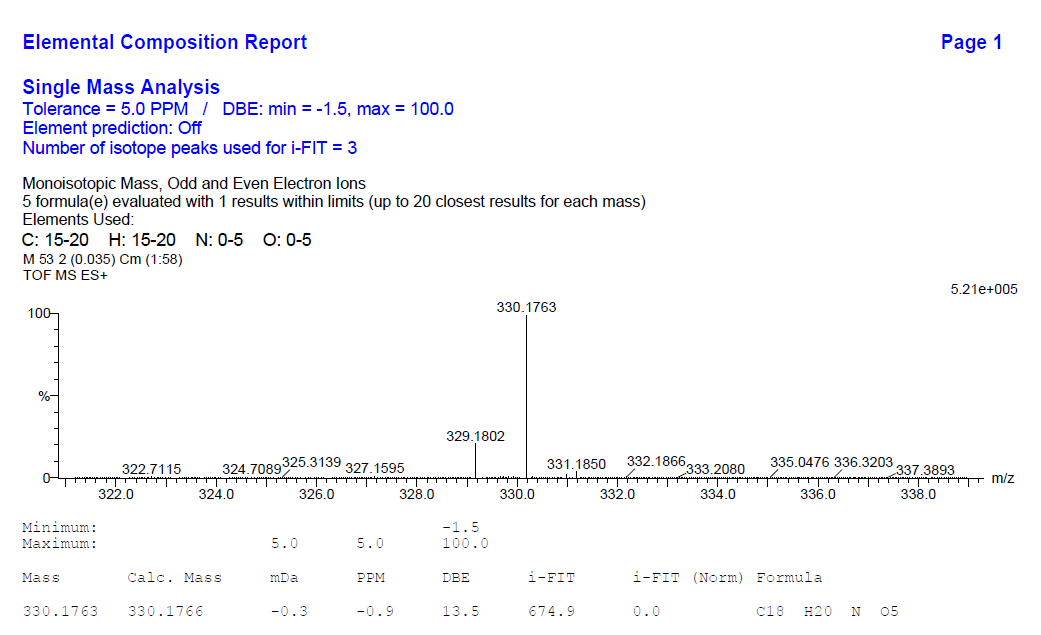
**

HRMS spectra of compound **4b**

**
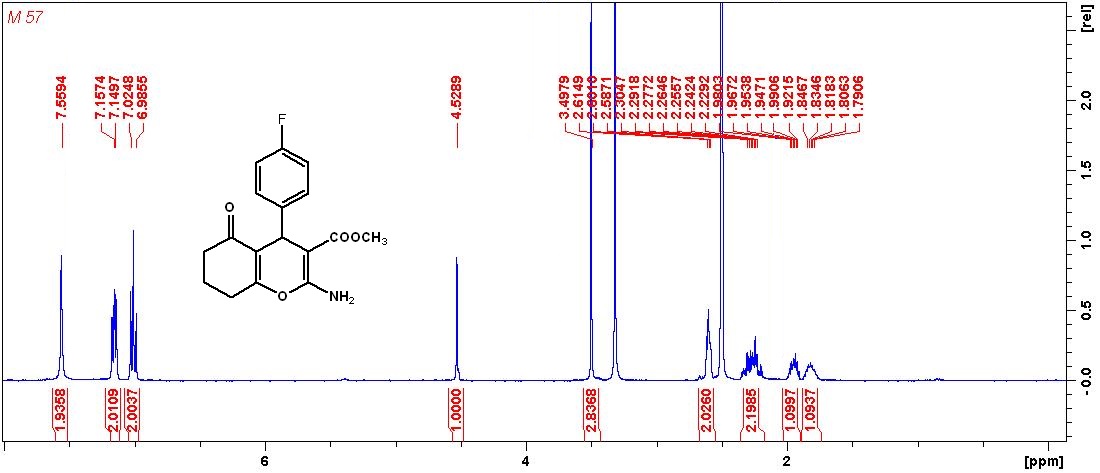
**

^1^H NMR spectra of compound **4c**

**
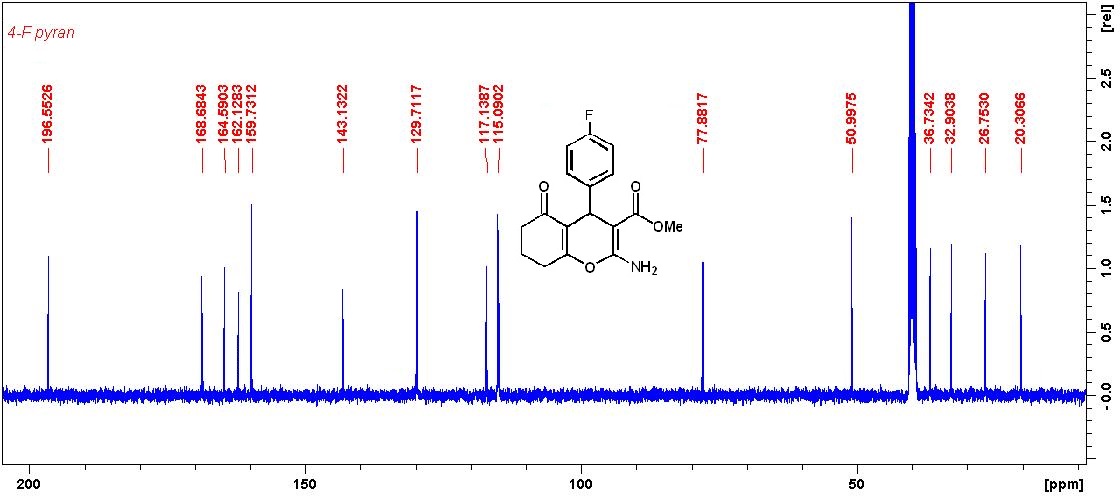
**

^13^C NMR spectra of compound **4c**

**
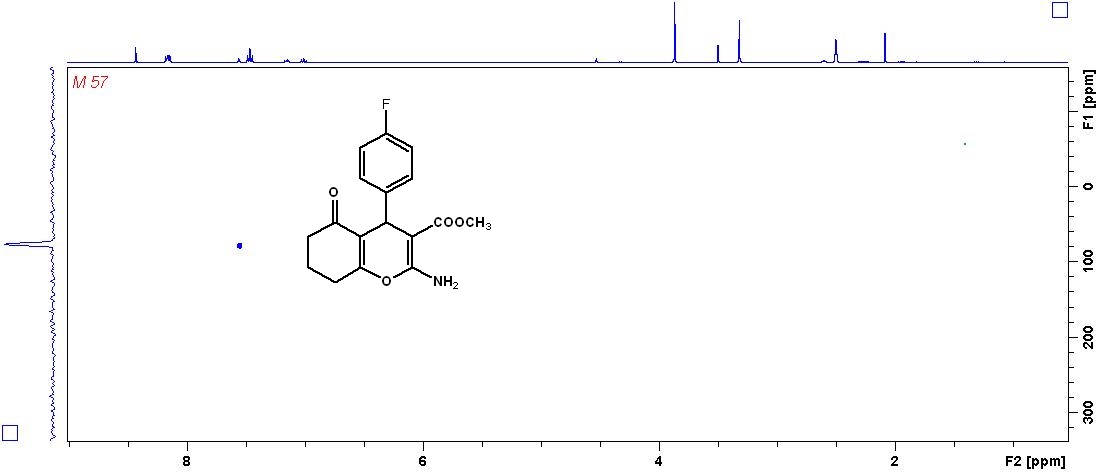
**

^15^N NMR spectra of compound **4c**

**
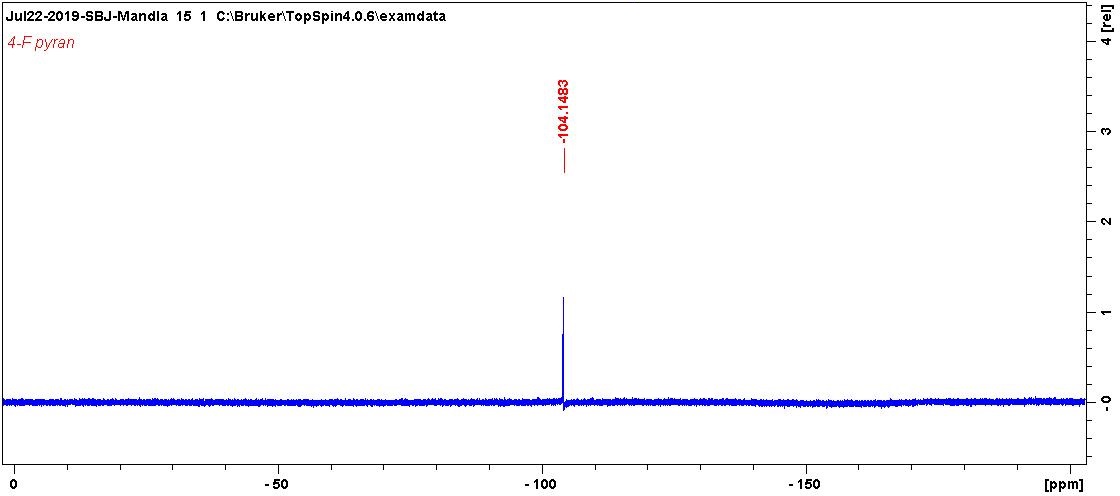
**

^19^F NMR spectra of compound **4c**


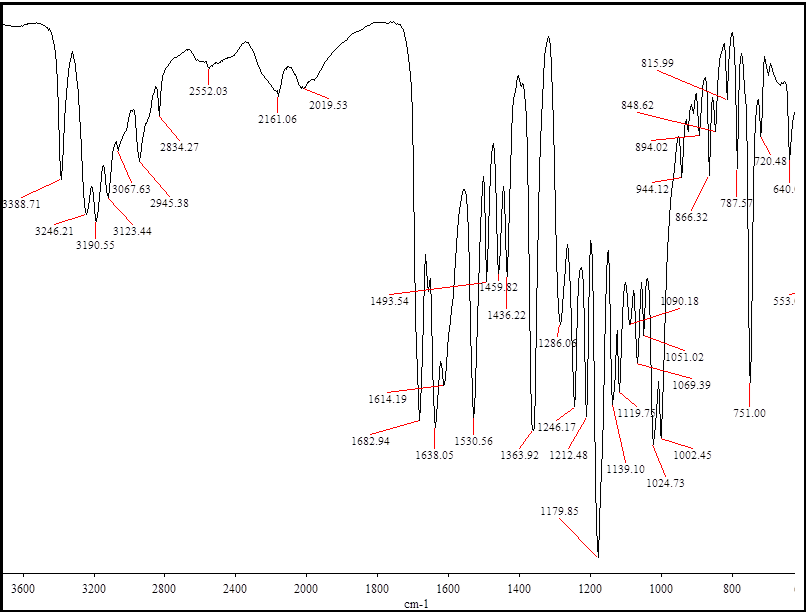
IR spectra of compound **4c**

**
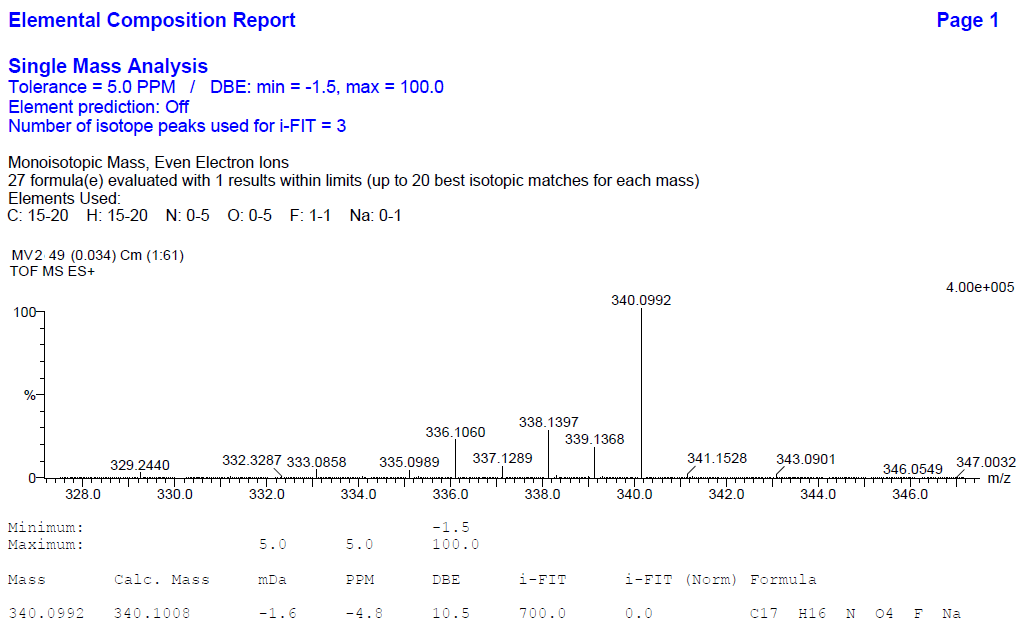
**

HRMS spectra of compound **4c**

**
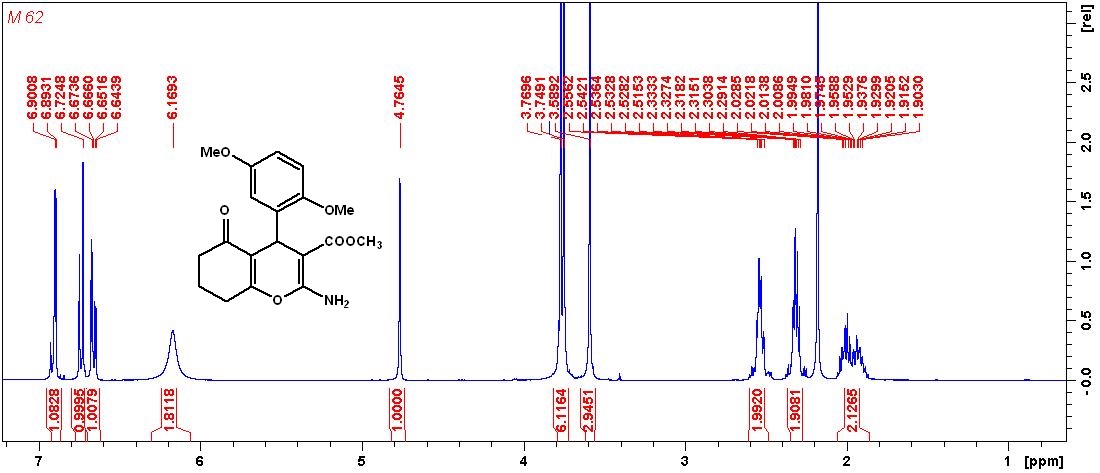
**

^1^H NMR spectra of compound **4d**

**
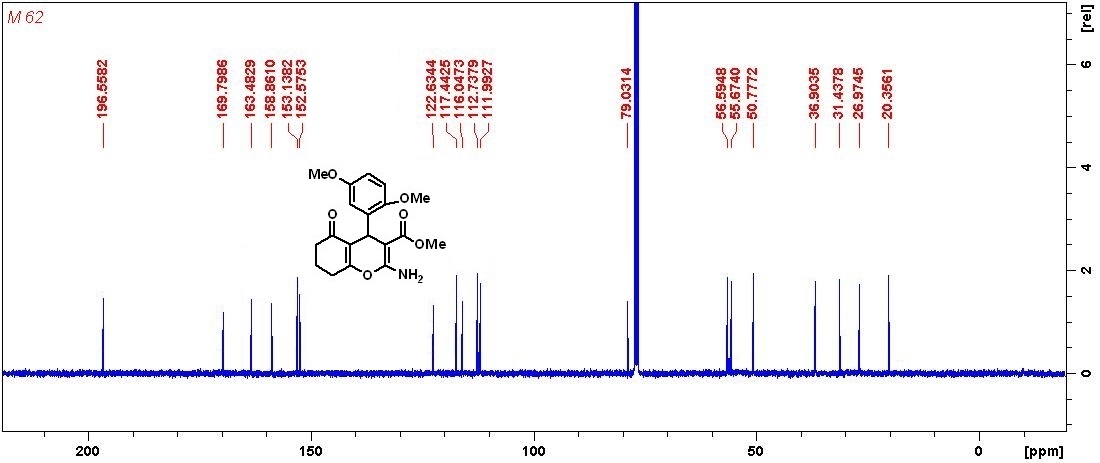
**

^13^C NMR spectra of compound **4d**


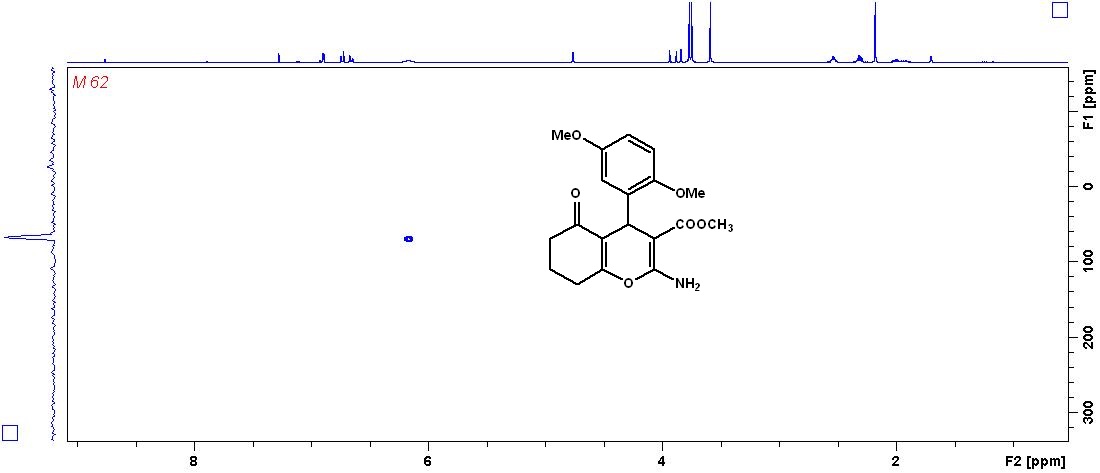


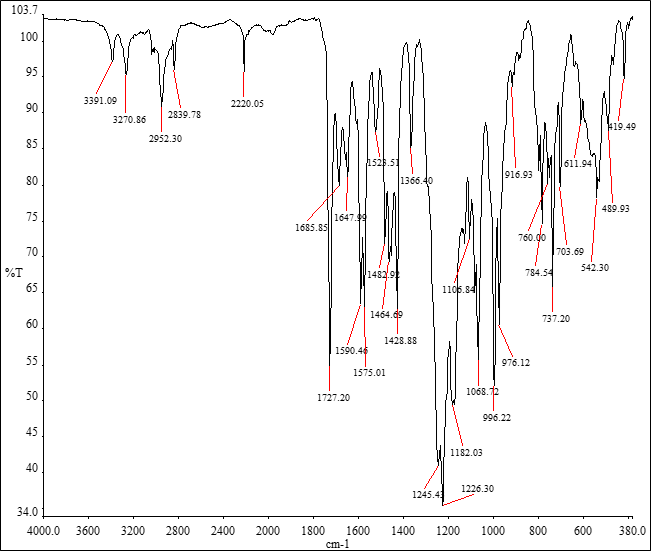
^15^N NMR spectra of compound **4d**

IR spectra of compound **4d**

HRMS spectra of compound **4d**


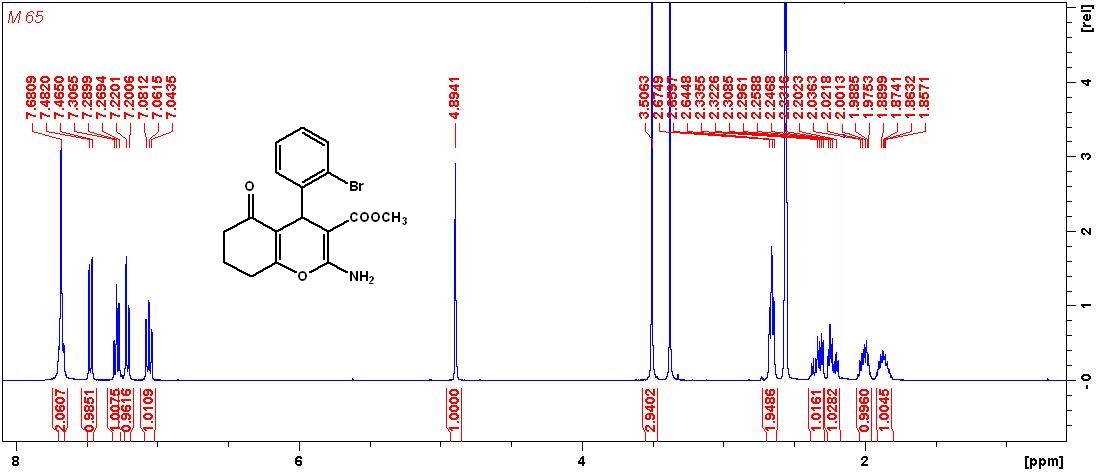


^1^H NMR spectra of compound **4e**


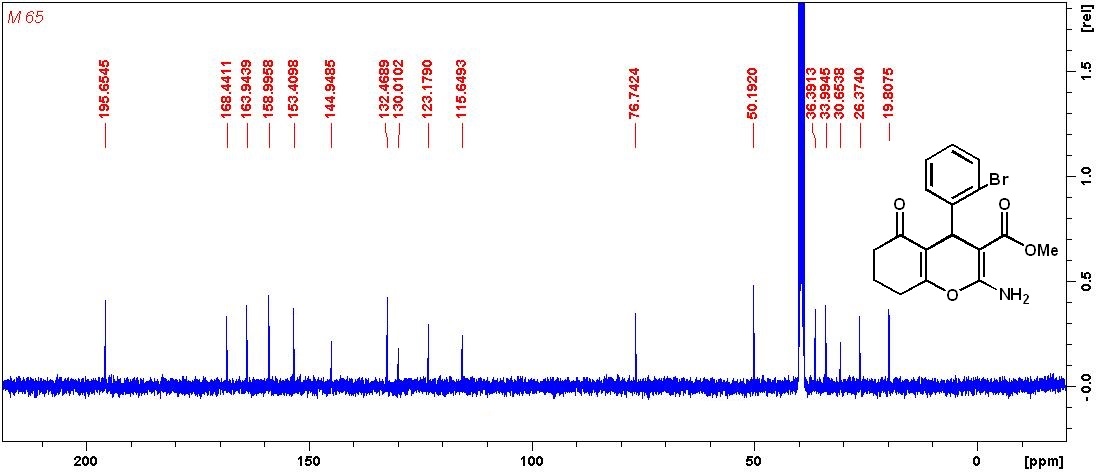


^13^C NMR spectra of compound **4e**


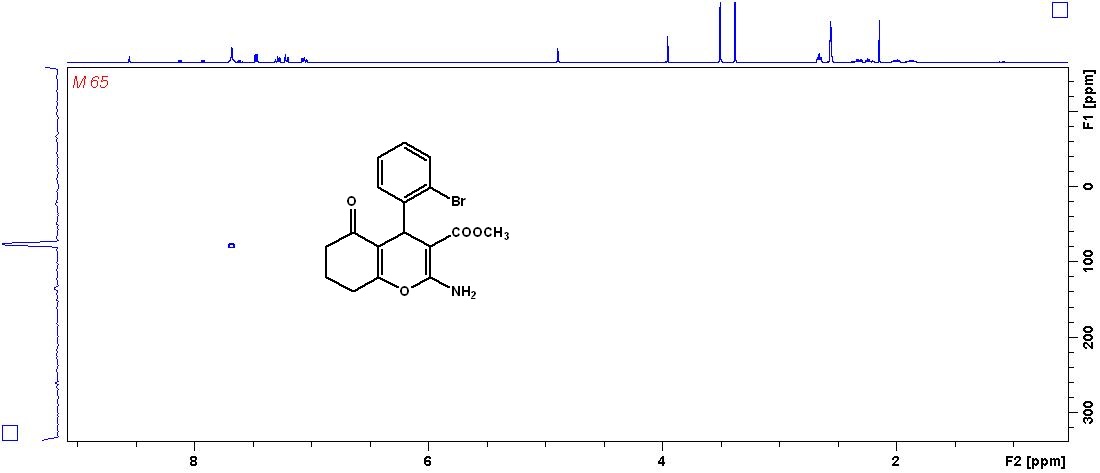


^15^N NMR spectra of compound **4e**

**
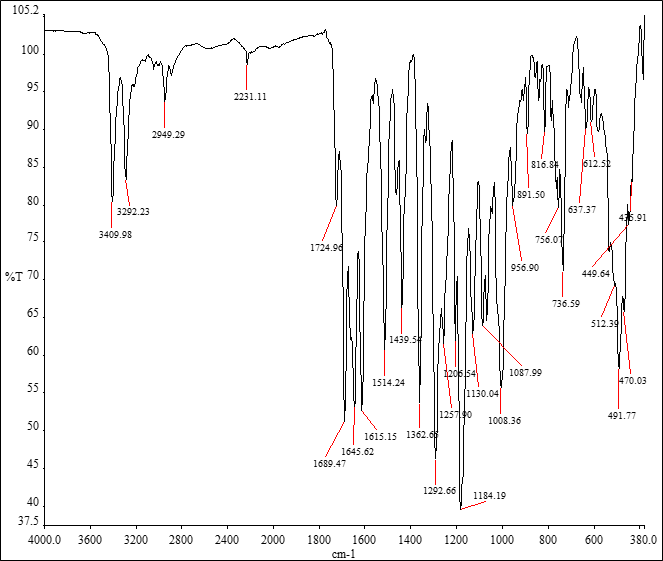
**

IR spectra of compound **4e**

HRMS spectra of compound **4e**


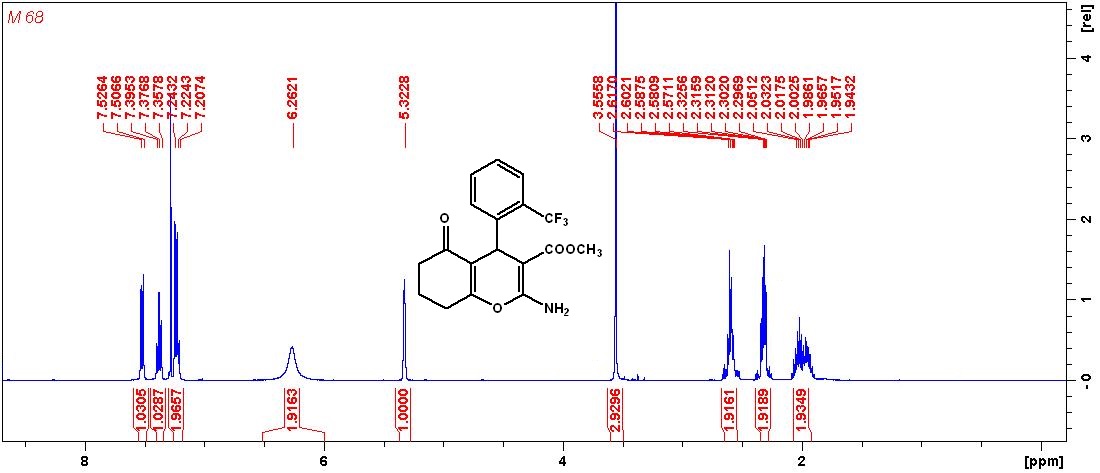


^1^H NMR spectra of compound **4f**


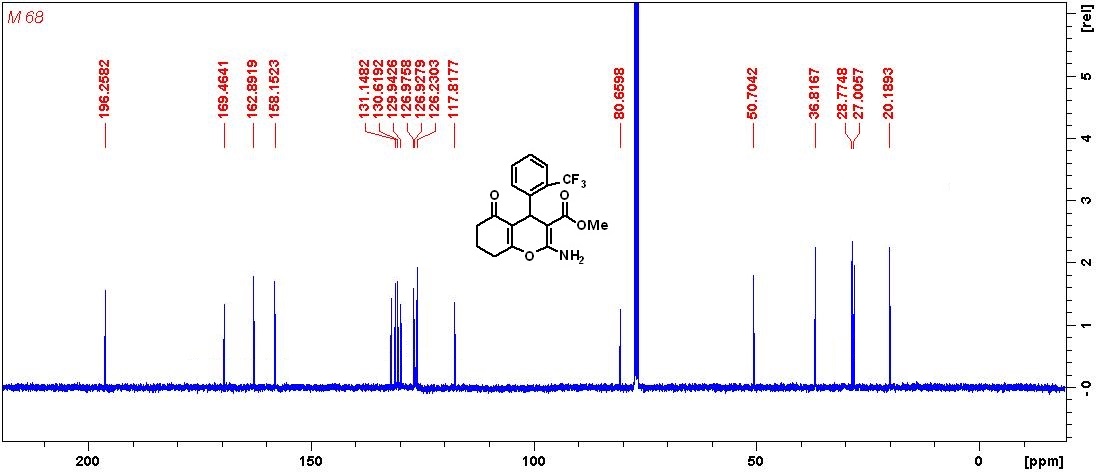


^13^C NMR spectra of compound **4f**


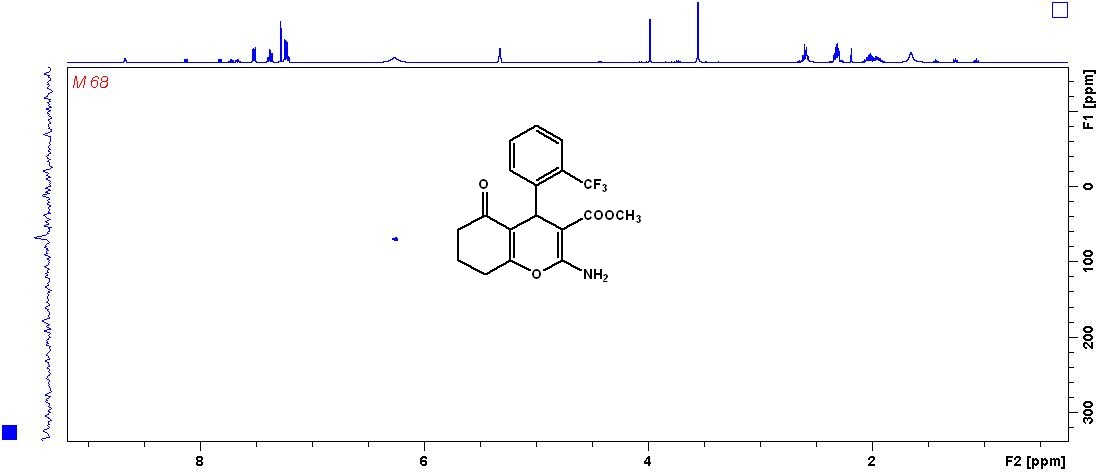


^15^N NMR spectra of compound **4f**

**
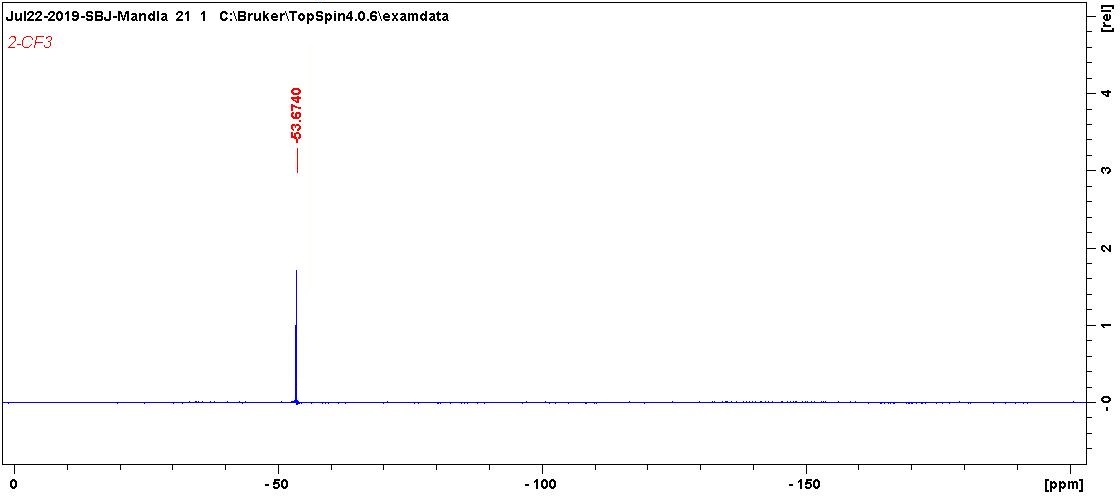
**

^19^F NMR spectra of compound **4f**

IR spectra of compound **4f**

HRMS spectra of compound **4f**


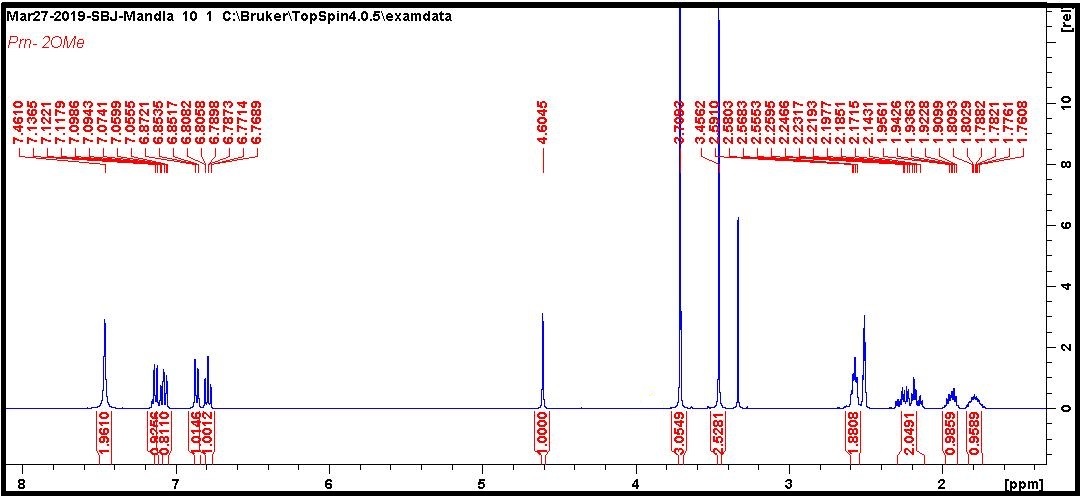
^1^H NMR spectra of compound **4g**


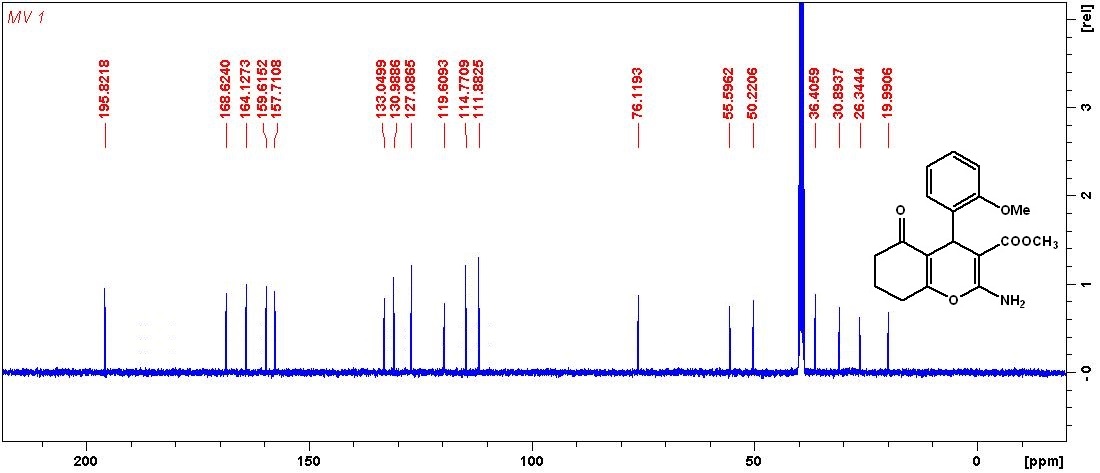


^13^C NMR spectra of compound **4g**

**Figure S1:** Selected HMBC interactions of –CH & a (1-6) Protons of 4g

**Figure S2:** ^1^H and ^13^C chemical shift of compound 4g


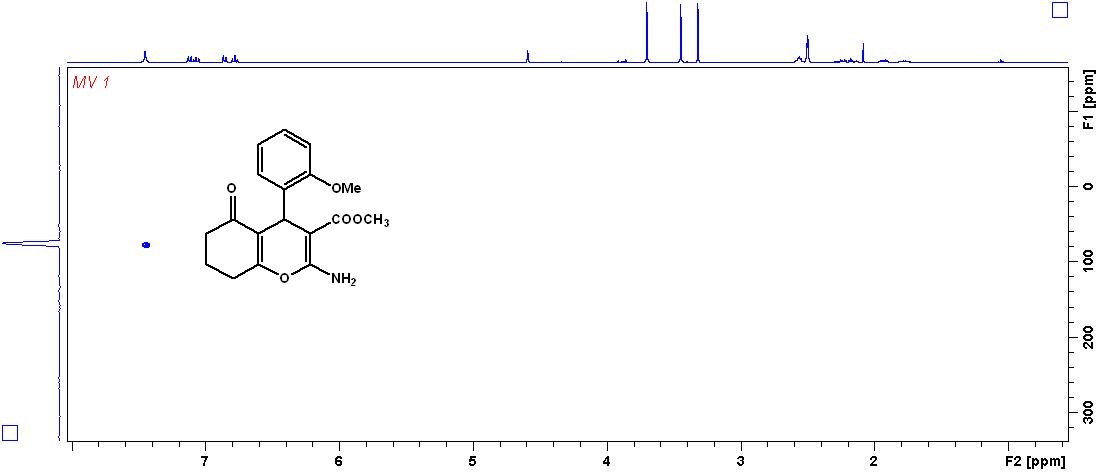


^15^N NMR spectra of compound **4g**

IR spectra of compound **4g**


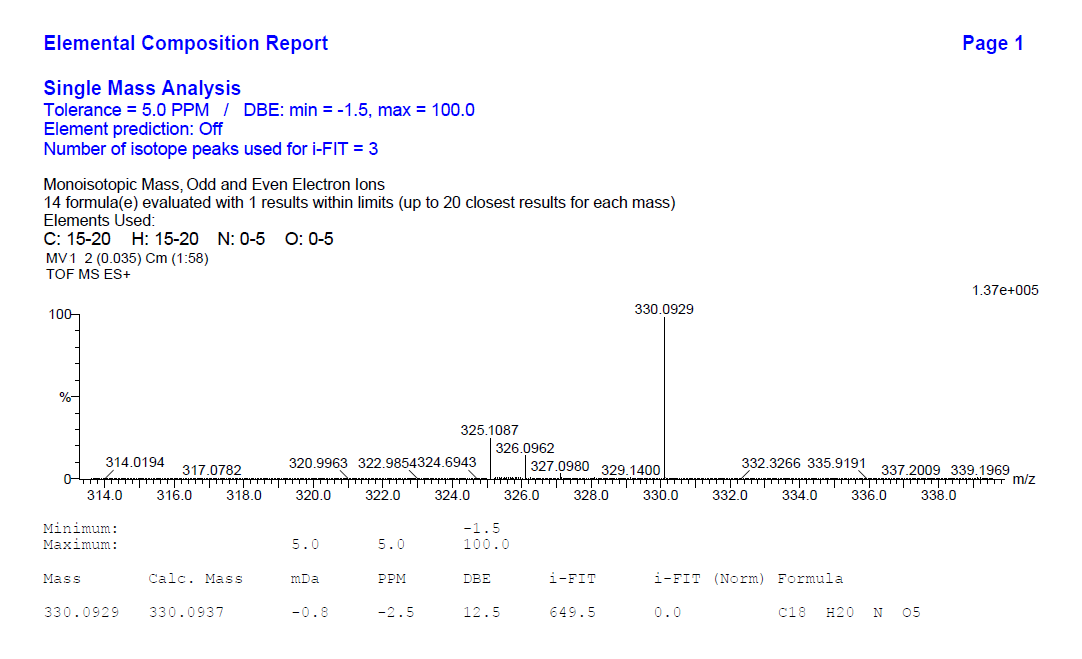


HRMS spectra of compound **4g**


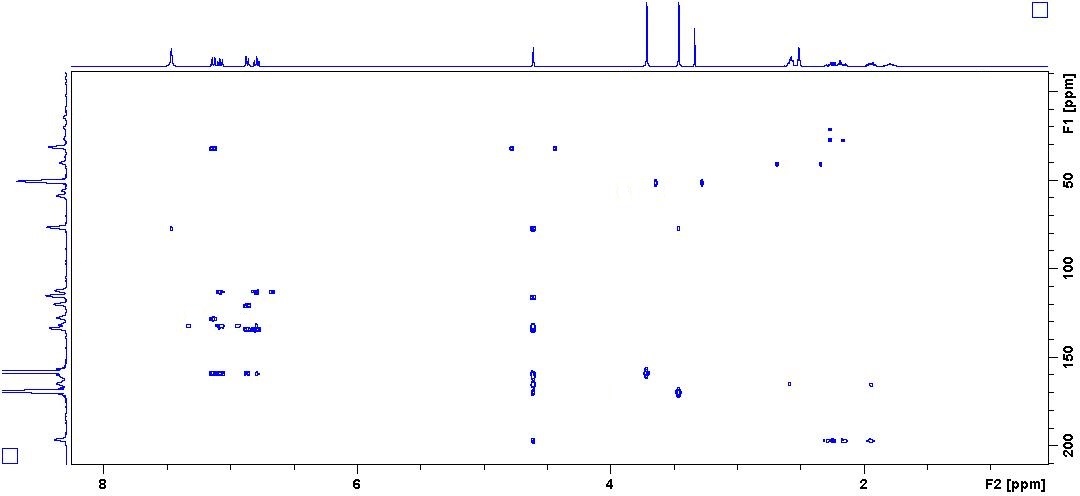


HMBC spectra of compound **4g**


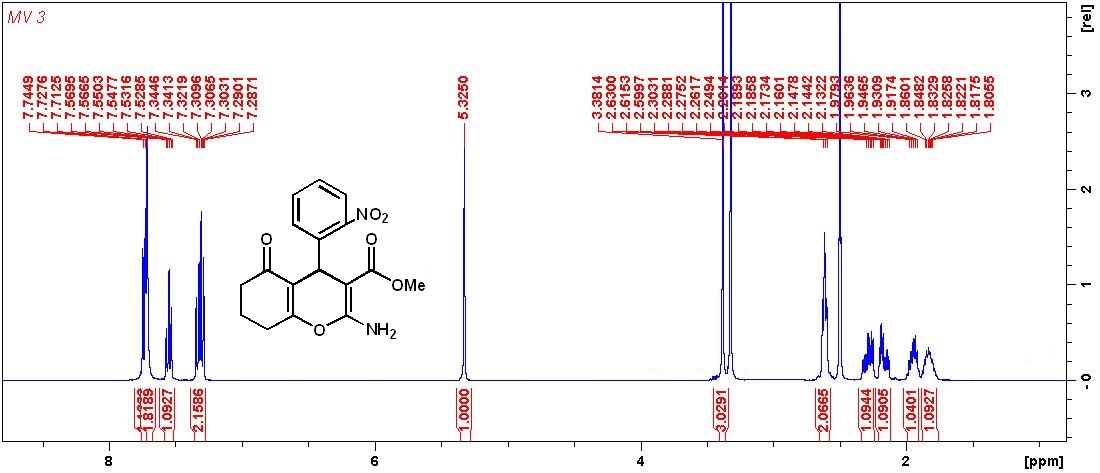


^1^H NMR spectra of compound **4h**


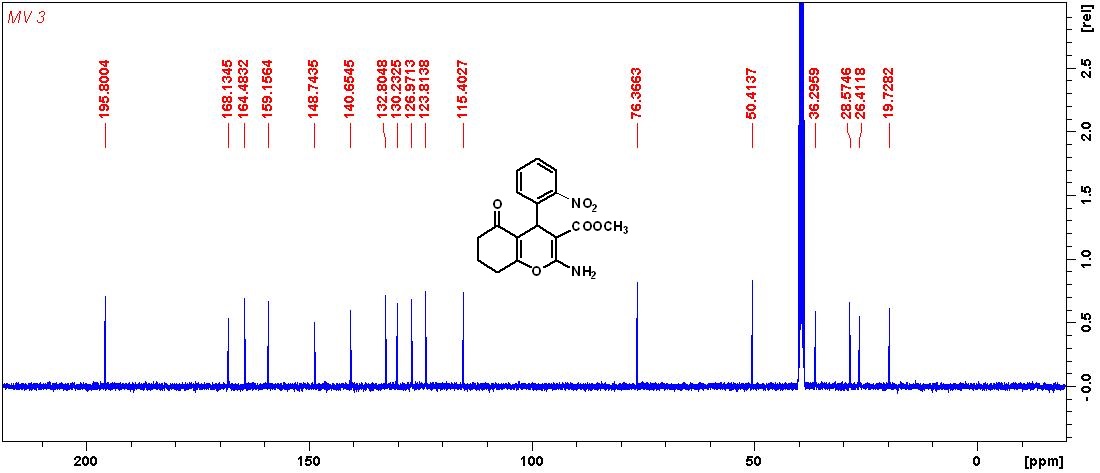


^13^C NMR spectra of compound **4h**


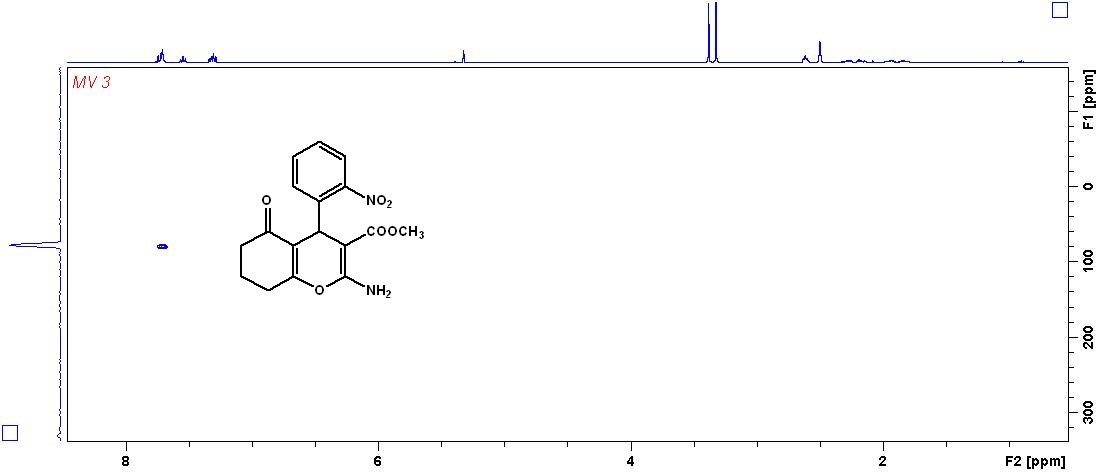


^15^N NMR spectra of compound **4h**

IR spectra of compound **4h**

HRMS spectra of compound **4h**


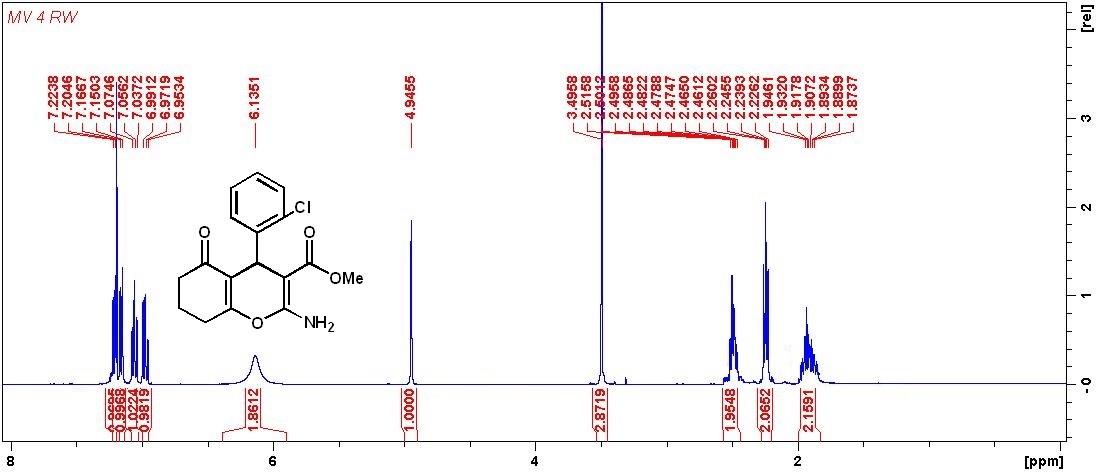


^1^H NMR spectra of compound **4i**


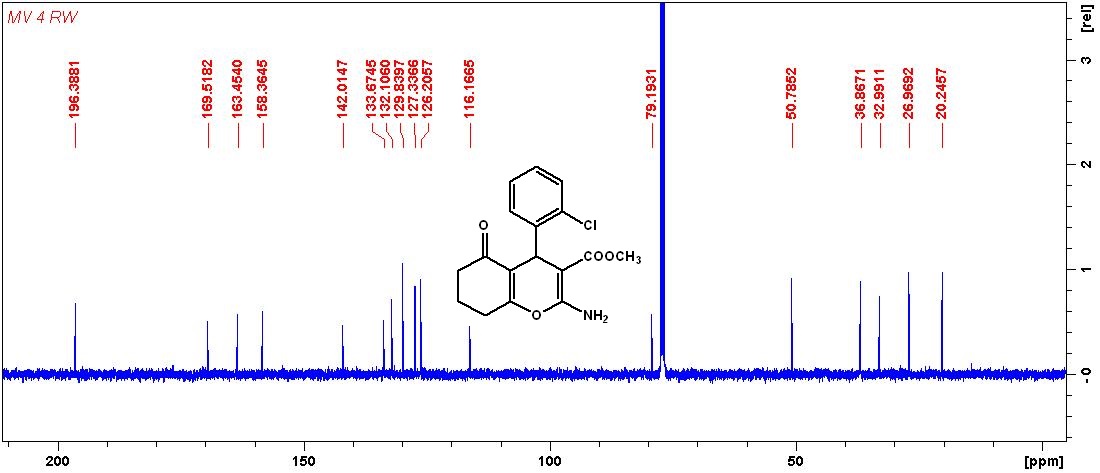


^13^C NMR spectra of compound **4i**


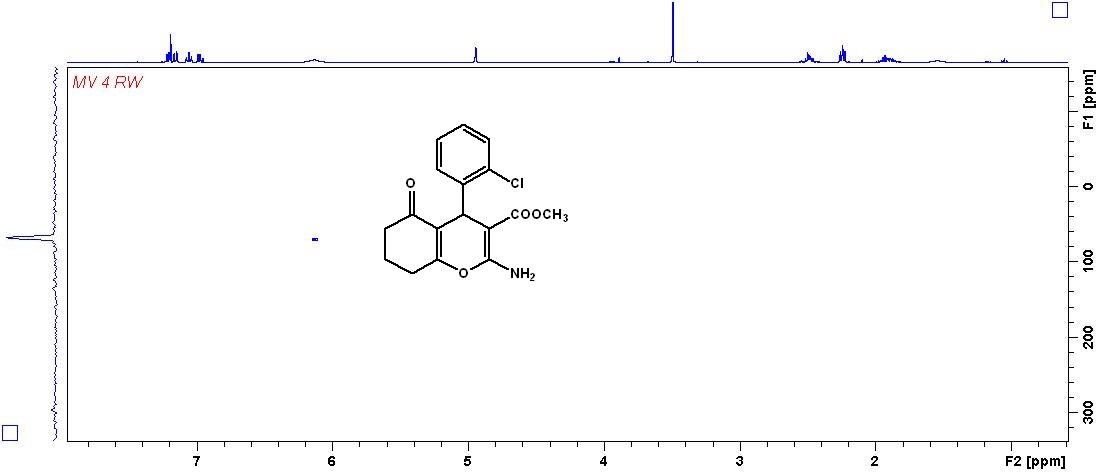


^15^N NMR spectra of compound **4i**

IR spectra of compound **4i**


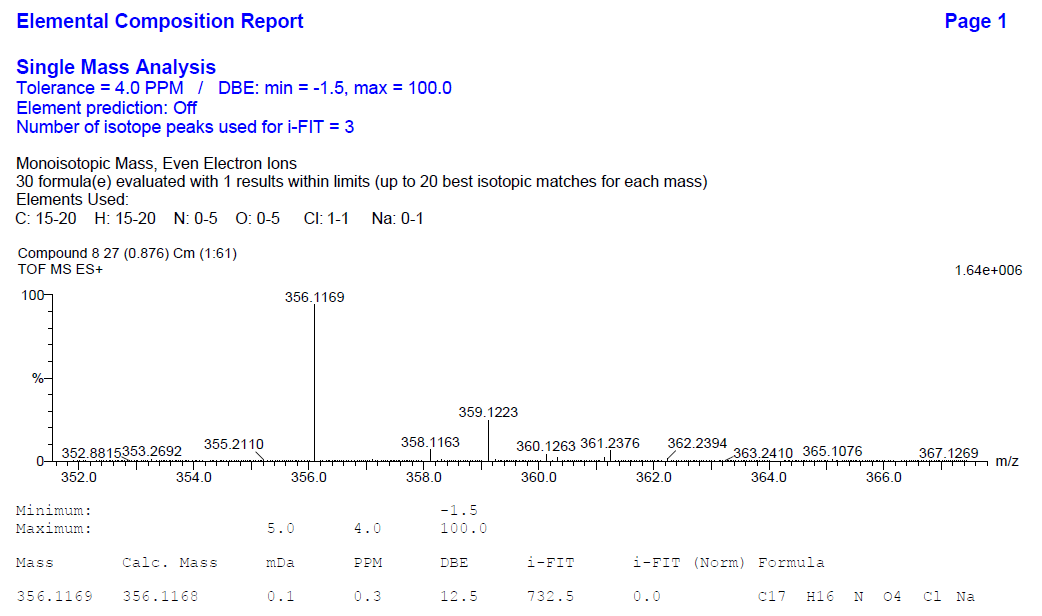


HRMS spectra of compound **4i**

**
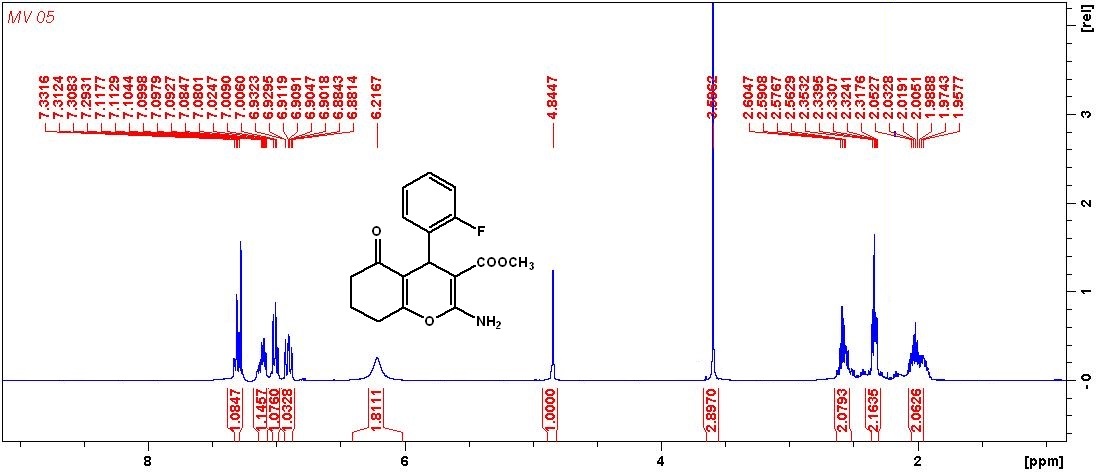
**

^1^H NMR spectra of compound **4j**


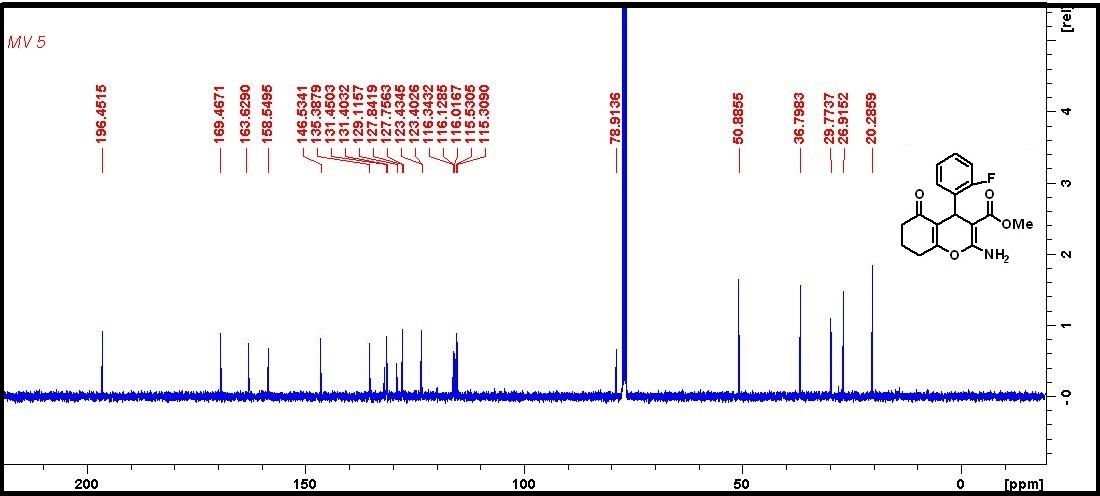


^13^C NMR spectra of compound **4j**


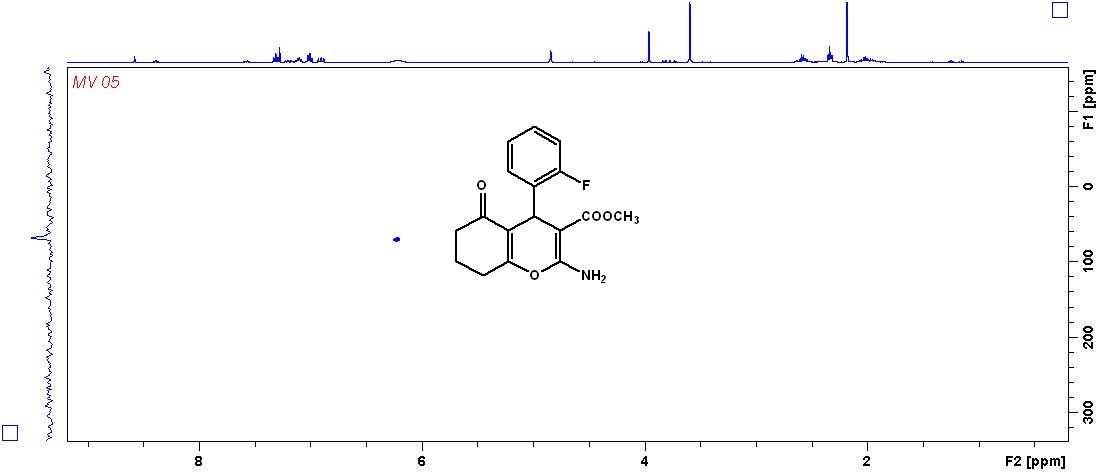


^15^N NMR spectra of compound **4j**


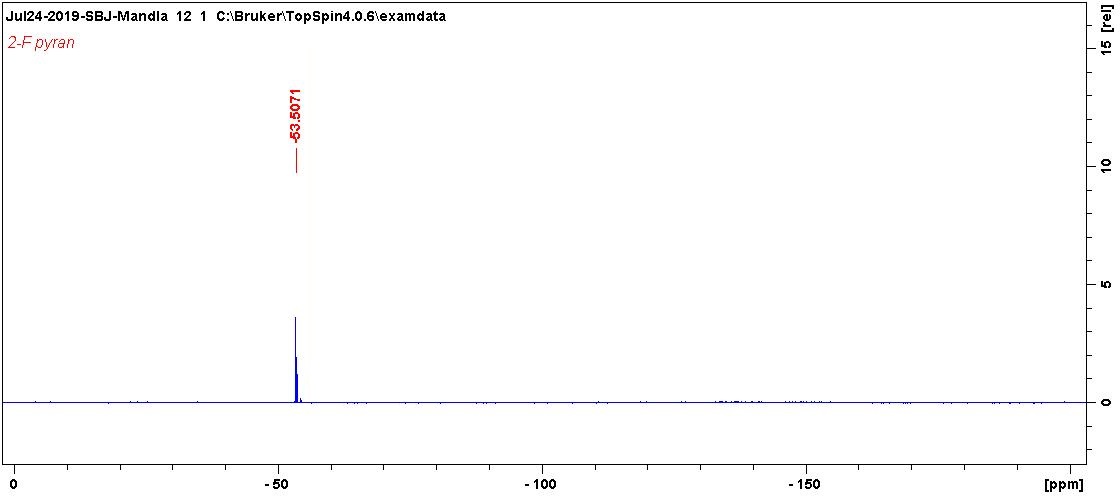


^19^F NMR spectra of compound **4j**

IR spectra of compound **4j**

HRMS spectra of compound **4j**


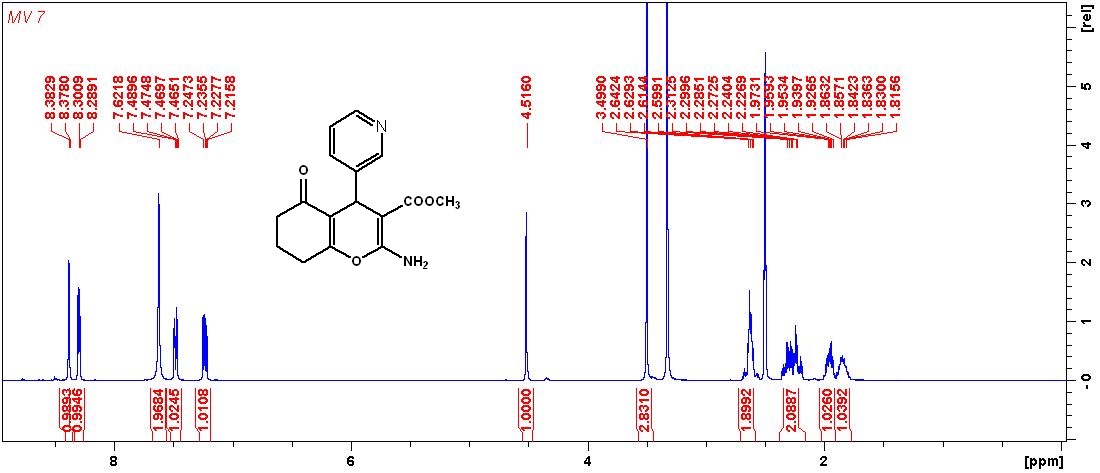


^1^H NMR spectra of compound **4k**


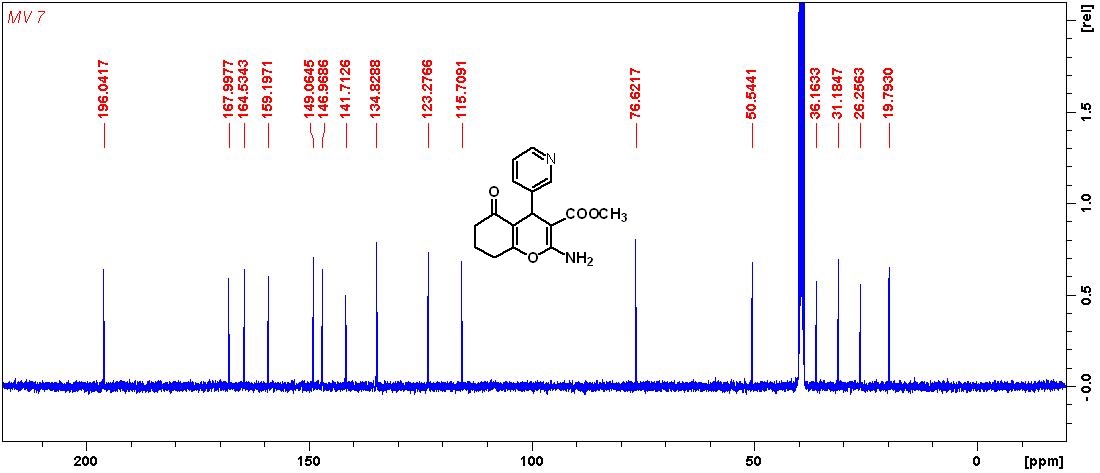


^13^C NMR spectra of compound **4k**


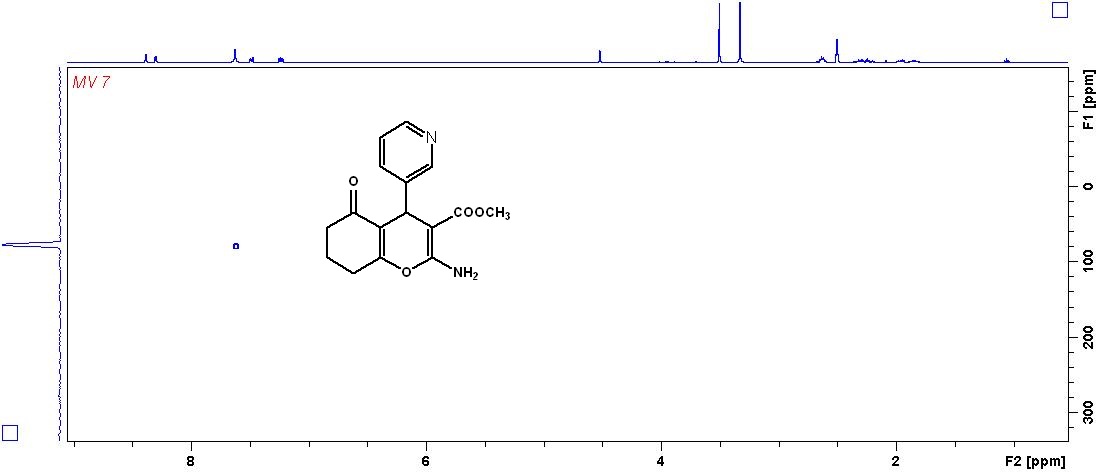


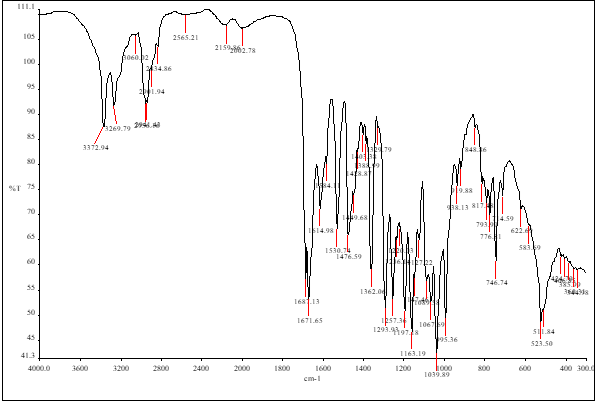
^15^N NMR spectra of compound **4k**

IR spectra of compound **4k**

HRMS spectra of compound **4k**

**Figure S3:** UV-Vis spectrum of Benzopyran compound

**Table S1:** Effect of various conditions for the synthesis of benzopyrans in presence of several catalysts.

| **Catalyst** | **Solvent** | **Condition** | **Time(m)** | **Yield%** | **Reference** |
| --- | --- | --- | --- | --- | --- |
| HTMAB | Water | RT | 180 min | 59-91 | 31 |
| H_2_PO_4_-SCMNPs | Solvent free | Heating 60 ^o^C | 15 min | 61-88 | 32 |
| Bmim[BF_4_] | Solvent free | Heating | 40 min | 78-89 | 33 |
| PPA–SiO_2_ | Water | Reflex | 10 min | 77-93 | 34 |
| Ca(OTf)_2_: Bu_4_NPF_6_ | Solvent free | Heating 120 ^o^C | 180 min | 55-92 | 35 |
| PbH(OH)_2_ 5mol% | EtOH/H_2_O | Reflex | 30 min | 41-88 | 36 |
| H_6_[P_2_W_18_O_62_].18H_2_O | EtOH/H_2_O | Heating 80 ^o^C | 50 min | 80-90 | 37 |
